# Pencil Graphite Electrodes: A Versatile Tool in Electroanalysis

**DOI:** 10.1155/2017/1905968

**Published:** 2017-01-31

**Authors:** Iulia Gabriela David, Dana-Elena Popa, Mihaela Buleandra

**Affiliations:** Department of Analytical Chemistry, Faculty of Chemistry, University of Bucharest, Panduri Av. 90–92, District 5, 050663 Bucharest, Romania

## Abstract

Due to their electrochemical and economical characteristics, pencil graphite electrodes (PGEs) gained in recent years a large applicability to the analysis of various types of inorganic and organic compounds from very different matrices. The electrode material of this type of working electrodes is constituted by the well-known and easy commercially available graphite pencil leads. Thus, PGEs are cheap and user-friendly and can be employed as disposable electrodes avoiding the time-consuming step of solid electrodes surface cleaning between measurements. When compared to other working electrodes PGEs present lower background currents, higher sensitivity, good reproducibility, and an adjustable electroactive surface area, permitting the analysis of low concentrations and small sample volumes without any deposition/preconcentration step. Therefore, this paper presents a detailed overview of the PGEs characteristics, designs and applications of bare, and electrochemically pretreated and chemically modified PGEs along with the corresponding performance characteristics like linear range and detection limit. Techniques used for bare or modified PGEs surface characterization are also reviewed.

## 1. Introduction

Graphite has both metallic and nonmetallic properties being thus useful as electrode material. According to Chehreh Chelgani et al. [1] 4% of the world graphite is used to produce pencils consisting of fine graphite powder in an inorganic (resin) or organic matrix (clay or a high polymer, e.g., cellulose). The pencil graphite leads are composite materials containing graphite (~65%), clay (~30%), and a binder (wax, resins, or high polymer) [2]. According to the European Letter Scale graphite pencils are marked with letters H (hardness) and B (blackness) and numbers indicating the degree of hardness or blackness from 9H (the hardest) to 8B (the softest). B type leads contain more graphite and are softer, and the harder H-type leads have more lead, whereas HB type pencil leads contain equal portions of graphite and clay [3–7]. Kariuki [5] showed that the added clay has an important influence on the chemical (e.g., ion exchange) and structural properties (e.g., degree of disorder and surface morphology) of the pencil graphite leads.

Aoki et al. [8] reported for the first time about the application of graphite reinforcement carbon (GRC), commonly employed as mechanical pencil leads, as voltammetric electrodes. The large commercial availability of GRC as graphite leads for mechanical pencils having low content of heavy metals impurities and high quality, controlled by the producer, along with all the other electrochemical features of the carbon-based electrodes led to the development of this type of disposable sensors.

Pencil graphite leads used as working electrodes are currently known as pencil graphite electrodes (PGEs). Besides the fact that they are cheap, PGEs are also easy to use and more convenient and there is no time-consuming electrode surface cleaning/polishing step. Applying different types of voltammetric techniques in order to quantify a variety of analytes from a wide range of samples, the used PGEs showed reproducible signals, yielding well-defined voltammetric peaks. These electrodes have proven to provide good sensitivity and reproducibility, being a viable, renewable, and economical tool.

Tavares and Barbeira [6] determined that most pencil leads, independent of the producer and hardness, have electrical resistance lower than 5 ohm, being thus suitable as electrode material. As a result of cyclic voltammetry (CV) studies, these authors also concluded that harder pencil leads present higher voltammetric signals and separation of the peak potentials are closer to the theoretical value for reversible systems. Despite the fact that Aoki et al. [8] reported that there were no differences between voltammograms of the [Fe(CN)_6_]^3−/4−^ and [Ru(NH_3_)_6_]^2+/3+^ redox couples recorded on GRC with different hardness, the nature of the pencil lead type influences the voltammetric response of the investigated analyte; for example, B pencil leads exhibited the best signal for the indirect determination of boron in the presence of tiron [4] and 6B leads were optimum for phenol and m-cresol differential pulse voltammetry (DPV) [9], whereas HB pencil leads were chosen for polyphenolic acids determination [10, 11]. Based on CV studies on K_4_[Fe(CN)_6_], Gong et al. [3] concluded that soft B type PGEs with large diameter enable an easy electron transfer generating thus higher signals and are proper for quantitative determinations, whereas small diameter H-type pencil leads provide better reversibility, being more adequate for qualitative investigations. According to Kariuki et al. [12] HB#2 graphite leads have electron transfer rates similar to glassy carbon electrodes (GCE).

Due to different interactions between an analyte and the common components of a graphite pencil lead, it is possible that electroactive species exhibit different voltammetric behavior on graphite pencil leads of the same hardness but are produced by various manufacturers [6].

As it was already mentioned, nowadays PGEs gain even more applicability in the electroanalysis of very different types of analytes from various matrices and not only. A careful literature search revealed that there are some reviews related to different types of carbon-based electrodes and different application areas of these electrodes and some of them mention also the PGEs [13]. A very recent review presented a history of the use of graphite pencils as electrodes and highlighted the application of PGEs in the analysis of environmental samples [14], but, to the best of our knowledge, there is no paper presenting an overview of general PGEs developments and applications. Therefore, this work is intended to review mainly the analytical applications of bare and modified PGEs along with their performance characteristics. A brief review of the methodologies of electrode surface modification in order to improve the sensitivity and selectivity of the detection method is also included. Moreover, practical examples of the most used techniques for surface characterization are given. It must be mentioned that a huge amount of literature data is related to PGE based biosensors and DNA-modified PGEs, but these types of sensors will constitute the topic of another review paper.

## 2. Discussions

### 2.1. PGE Characteristics and Comparison to Other Carbon-Based Electrodes

The electrode material of the PGE is represented by a general purpose writing pencil offering the advantages of being user- and environmental-friendly (it is nontoxic), largely commercially available, and simple to purchase at low costs (e.g., in Romania the graphite pencil lead used for one PGE costs less than 0.04€), being thus a disposable and readily renewable electrode. According to Kariuki et al. [12] the cost per electrode is approximately at least 190$ for GCE and 0.13$ for PGE.

The GRC contained in pencil leads is not as rigid as glassy carbon (GC) but it is more rigid than carbon paste (CP) or pyrolytic carbon (PC) [6, 8].

Like the other carbon-based working electrodes (GC, PC, CP, or CF (carbon fiber)) the PGE presents high (chemical and mechanical) stability, a large working potentials window (e.g., −0.8 to 0.8, −1.0 to 0.8, and −0.8 to 0.6 V versus SCE in H_2_SO_4_, KCl, and NaOH solutions, resp.), which could be extended to more negative values in comparison to metallic (Au or Pt) electrodes [8]. Perdicakis et al. [15] investigated the accessible electroactivity ranges of PGE in different media and the electron transfer rates of model redox couples [Fe(CN)_3_]^3−/4−^ and [Ru(NH_3_)_6_]^3+/2+^.

The reproducibility of the voltammetric determinations on solid electrodes is highly dependent on the reproducibility of the electrode active surface, usually ensured by cleaning/polishing/pretreatment of the surface before each recording. These are time-consuming steps which also may destroy the crystalline structure of the graphite. These drawbacks can be eliminated by using disposable graphite pencil leads which can be easily and rapidly replaced, shortening thus the analysis time. Due to the strictly controlled quality of the pencil leads during their manufacturing, uniform composition and surface are provided and the analytes voltammetric signals recorded on individual PGEs of the same type are very similar resulting in a good electrode-to-electrode reproducibility [8]. Therefore, PGEs provide a convenient surface renewal which leads to a good reproducibility of the voltammetric determinations [16]. Even in the early period of use, PGEs represented by GRC proved a good reproducibility, durability, and stability [8, 17].

According to Kariuki et al. [12] more reproducible results (as indicated by the percent relative standard deviations (RSD%) of the peak potentials and areas) were obtained in electrochemical studies on ferricyanide and gallic acid and for the DPV determination of antioxidants in various samples when a disposable wooden PGE was used instead of a CGE or a mechanical PGE, because all the measurements performed during the entire experiment were made on the same HB#2 pencil lead. Vestergaard et al. [18] presented RSD% for the peak potentials and peak heights of <5.0% and <10.0% obtained for eight flavonoids and of 1.7% and 3.2% for a copper solution, respectively.

The reproducibility and stability of the signals showed by the PGEs in correlation with the lead length (until 4 mm) were studied, obtaining very good results for all the lengths [19].

Recently, a study related to the voltammetric determination of strontium ranelate using an unmodified PGE indicated a good reproducibility of the results, similar or even better to those for a CPE and a GCE [20].

Özcan and Şahin [21] reported good fabrication reproducibility (RSD 2%) for the electrochemically treated PGEs (ETPGEs). Intra- and interday precision of the chlorpromazine DPV analysis using an ETPGE were expressed as percentage relative standard deviation of 2.2 and 4.8%, respectively [22].

Bond et al. [23] reported that PGEs give small background currents generated by the clays and polymers contained in the pencil lead but, nevertheless, these currents are lower than those presented by the conventional solid electrodes, like glassy carbon, gold, and platinum [24], but higher than those of boron doped diamond electrodes [25]. Several authors enumerate among others the low background current as an important feature of the PGEs [19, 26–33]. According to Wang et al. [16], the nongraphitic components of PGEs do not give any background signal, but the different types of pencil leads with different compositions of polymers and clays, besides graphite, and roughness have different response characteristics, the most advantageous being HB pencil leads, generating a high analyte (i.e., guanine) signal and relatively low noise. Thus, even if PGEs present background currents, they can be corrected by the advanced data processing programs of the computerized instruments leading to good signal-to-noise ratios [25].

Due to their large active electrode surface area (e.g., 0.255 cm^2^ for PGE compared to 0.0951 cm^2^ for CPE [26]), the PGEs can be used to analyse low concentrations and small volume samples without any preconcentration/deposition step, reducing thus the analysis time [18]. They can also be miniaturized and easily modified [34].

The high electrochemical reactivity of PGEs may be due to the irregular pencil graphite surface morphology which may enhance the active surface area of the electrode, whereas the edge plane sites on the surface result in a better electron transfer and a higher resistance to surface passivation [35]. Comparing voltammograms recorded on PGE with those obtained on GCE some authors attributed the higher peak intensities obtained on PGE to the clay [5] that generates a porous structure and a high specific surface area of the pencil graphite and to a good electrocatalytic effect of the pencil graphite on the electrode process of some analytes (e.g., acyclovir [27], eugenol [36], and niclosamide [37]).

Different papers reported comparisons between the performances of PGEs and other electrodes related to the electroanalysis of different compounds. The lead of a mechanical pencil, that is, GRC electrode, exhibited a well-defined reduction peak of ozone in acidic media, presenting better performances than GC, Au, or Pt for the detection of this analyte [38], whereas the boron voltammetric signal is six times higher and has a lower oxidation potential on PGE than on GCE [4]. Using square wave voltammetry (SWV), Kawde [39] reported that a Bi-GCE presents better sensitivity than Bi-PGE for the simultaneous determination of Pb(II), Cd(II), and Zn(II) from water samples, but Bond et al. [23] demonstrated the better performance of the “low tech” renewable PGE with respect to the “high tech” GCE by comparing the data obtained for cadmium and lead (ng·mL^−1^ range) stripping voltammetric analysis on in situ mercury film coated electrodes, whereas 5H pencil leads are used as working electrode for the acetaminophen detection in a home-made flow-through electrochemical cell performed similarly with the GCE [40]. Rubianes and Rivas [41] compared the performances of different carbon-based electrode materials, among them being graphite as PGE, as support for a melanin type polymer sensor developed for the dopamine amperometric determination. The proper electrode material was GC, whereas electrodes based on graphite (i.e., pencil graphite and graphite paste) were not suitable for this task, probably due to their inherent high electroactivity. CV studies performed on a ferricyanide solution using GCE, mechanical PGE (M-PGE), and wooden PGE (W-PGE) emphasized that W-PGE behaves similar to GCE and superior to M-PGE in terms of electron transfer kinetics. With respect to SWV analysis of gallic acid (GA) all electrodes demonstrated similar linearity, but the W-PGE exhibited a higher sensitivity. Despite the fact that the limits of detection (LD) obtained with the investigated electrodes are of the same order of magnitude, the lowest LD, 2.93 · 10^−4^ M GA, was obtained using M-PGE. The applicability of W-PGE as an antioxidant sensor was tested on fruit and vegetable extracts, hot beverages, dopamine, and uric acid, presenting in all cases more intense peaks than GCE [12]. CV measurements of ascorbic acid emphasized that PGE gives similar results with those obtained with more expensive carbon electrodes [42].

Adenine and adenine-copper complexes present higher signals on PGE than on CPE along with a peak shift towards lower potentials and the electrolyte discharge appearing at higher positive potentials enabling thus more sensitive determinations and indicating an easier electrode process and a larger potential range for voltammetric measurements, respectively [43].

Cyclic voltammetric peaks of vitamin B1 and B6 were more intense when recorded on PGE than on GC and Pt electrodes [44] and those of famotidine have about 1.4 times higher sensitivities on PGE compared with GCE [45]. Our studies revealed that polyphenolic acids presented the best shaped and most intense oxidation peaks on PGE in comparison to GC and Pt electrodes. The high sensitivity of the DPV on the PGE determination of these antioxidants was also emphasized by the attained LD (i.e., 8.83 · 10^−8^, 7.93 · 10^−9^, and 7.14 · 10^−8^ M for caffeic [10], rosmarinic [11], and chlorogenic acid [46], resp.), which are among the lowest LDs reported in the literature for other similar studies. *α*-Naphthol gave a high, well-defined peak at PGE, whereas it did not respond well at GC, CP, Pt, and Au electrodes. Electrochemical treatment of PGE increased the sensitivity of the determination resulting in a very low LD of 1.5 nM [47].

The higher sensitivity of PGE in comparison to CPE and GCE was also demonstrated by the LDs obtained for strontium ranelate of 0.17, 0.24, and 0.39 *μ*g·mL^−1^ for peak 1 and 0.19, 0.27, and 0.51 *μ*g·mL^−1^ for peak 2, respectively [20]. The LD and limit of quantification (LQ) of dantrolene sodium obtained at PGE (0.052 and 0.158 *μ*g·mL^−1^) and GCE (0.095 and 0.289 *μ*g/mL) [48] indicate a more sensitive determination on PGE. Other proofs of the high sensitivity of the voltammetric determinations using PGE as working electrode are the low LDs and LQs values recorded for flutamide (1.55 · 10^−8^ M and 5.16 · 10^−8^ M), irinotecan (1.68 · 10^−9^ and 5.69 · 10^−9^ M) [49], capsaicin (3.7 · 10^−9^ M and 1.2 · 10^−8^ M), dihydrocapsaicin (9.1 · 10^−10^ M and 3.0 · 10^−9^ M) [25], and paclitaxel (2.46 · 10^−9^ M and 8.23 · 10^−9^ M) [26], respectively.

### 2.2. Designs of PGE

The most commonly used design of a PGE consists in a pencil lead introduced in a commercially available mechanical pencil acting as holder. A certain length of the lead remains outside the pencil. In order to maintain a constant electroactive surface area the pencil holder is kept in upright position and always a constant length of the pencil rod is introduced in the solution to be analysed [10, 11, 21, 24, 25, 43–45, 49–61]. Other authors reported the direct connection of the PGE to the potentiostat cable [62] or the mounting of the PGE on an improvised electrode clip [63]. One paper describes the sealing with epoxy of the graphite lead into a syringe acting as holder. 1 cm of one lead end remained outside the holder, whereas at the other end a copper wire was attached [28]. In other studies a pencil lead graphite disc electrode is used, being prepared by introducing the pencil lead into a Teflon tube [29, 64–67], a PVC tube [68], and a tip of a micropipette [35] or by covering its outside surface with Teflon band [2, 69] or with a nonconductive parafilm [9] so that only the cross-sectional surface remains free. The electrical contact between the electrode and the instrument was realized through a metal wire wrapped at the other end of the pencil lead. Bund et al. [70] described a PGE developed by connecting a silver wire to the graphite lead and the whole system was introduced through a silicon rubber into a glass tube so that the lead can be pushed out and its surface renewed by polishing before each recording. For clozapine analysis in human plasma a microsystem consisting of a micropipette tip was obtained by inserting the electrodes, with PGE as working electrode, through a septum [71]. Graphite pencil lead microelectrodes (diameter ~ 12 *μ*m) employed for cell dielectrophoresis were obtained by electrochemical etching of 0.5 mm HB leads in 1 M NaOH. The obtained microelectrode was insulated by an electrogenerated o-phenylenediamine polymer or it was embedded in a micropipette tip by melting [72].

More recently, flexible paper based analytical electrochemical devices (PADs) were developed. Paper is the substrate on which the electrodes are drawn with graphite pencils [30, 73–84]. This strategy is simpler and cheaper than lithography [85].

### 2.3. Applications of PGE

Due to their inherent electrochemical and economical characteristics PGEs found in recent years a widespread application in various fields. In the field of electroanalysis, bare (Table 1) or modified PGEs (Tables 2–4) have been applied to the determination of both organic and inorganic compounds from different samples ranging from quite simple matrices like water till complex biological fluids like human urine or serum.

In 1996, pencil leads were used as new electrodes in abrasive stripping voltammetry [86]. Two years later a vibrating electrode based on a mechanical graphite pencil was reported to be used for the simultaneous potentiometric stripping determination of Cu(II), Cd(II), and Zn(II) traces in small samples [87]. Perdicakis et al. [15] proposed the use of pencil graphite electrodes for the immobilization and characterization of different types of electroactive species using voltammetry on microparticles. Later on, this principle was applied to get information about lead corrosion in archeological samples [88] and the composition and the degradation products of archeological lead. The electrochemical authentication was based on the observation of stripping peaks for patination products metals present in ancient lead, the potential shift of the reduction peaks, and signals due to the PbO_2_ reduction [89].

GRC existing as mechanical pencil leads constituted the working electrode for a dopamine determination in the range 5–120 *μ*M in the presence of 5 to 20 times higher ascorbic acid concentrations [17].

A bare PGE was employed for the rapid DPV determination of total polyphenolic content of Sea Buckthorn leaves extract [191] and also for the electrochemical study of flavonoids and their interaction with copper ions. The obtained results could be useful for understanding the flavonoids role in decreasing the copper-induced oxidation of the substrate [18].

#### 2.3.1. Electrochemical Pretreatment of PGE

The electrochemical characteristics of a PGE, which depend on its surface properties, can be changed/improved either by pretreatment or by chemical modification of the electrode active surface. The electrochemical pretreatment of a PGE, which consists in the electrooxidation or electroreduction of the PGE in the selected conditions, is the most rapid, simplest, cheapest, and eco-friendly (no use of toxic reagents) approach in comparison to other surface modification strategies [54, 106]. It was shown that electrochemical pretreatment activates the electrode surface by modifying its surface and redox characteristics [2, 21, 57, 106]. In comparison to nontreated PGE, the electrochemically pretreated PGE (ETPGE) presented improved electrocatalytic activity (e.g., for uric acid and ascorbic acid [2], methamphetamine [101], sulfide [192]), higher sensitivity (e.g., a 2–5 times higher sensitivity for monophenols [9] and a 40-fold increase of uric acid voltammetric signal [91]), higher selectivity by increasing the peaks separation [2, 21], and better adsorption capacity of organic compounds [57, 106, 192, 193]. This properties strongly depend on the activation conditions, that is, electrochemical procedure, electrolyte (type and concentration), applied potential or potential range [54], number of scans, and scan rate [21, 54, 106].

PGEs were pretreated potentiostatically by maintaining the electrode in an electrolyte for more than 30 s at a selected potential higher than 1.3 V [9, 53, 55, 56, 60, 70, 99–101, 105, 192, 193] or by scanning the potential at a given scan rate, for a certain number of cycles within a large potential range [2, 57, 58, 61, 69, 103, 106, 188, 194]. Some papers report a comparison between the two mentioned activation procedures and depending on the electrochemical performances obtained for the investigated analyte one of them was selected; for example, chronoamperometric activation of PGE was used for alkylphenols [50] and tannic acid [51] analysis and cyclic voltammetric activation of PGE for dopamine [21] and bisphenol A [54] detection. A precharged PGE developed by charging the electrode surface using CV in a NaOH solution was applied to phenols sampling and determination [104].

The performances of ETPGE depend on the supporting electrolyte used during the electroactivation and on the analyte; for example, PGE pretreated in H_3_PO_4_ had higher sensitivity towards dopamine and ascorbic acid, whereas the same electrode activation performed in a mixture of LiClO_4_ and Na_2_CO_3_ led to the best sensitivity and selectivity for uric acid [106] and PGE electroactivated in a mixture of LiClO_4_ and NaOH presented the best performances for bisphenol A detection [54].

All these electrochemical improvements of ETPGE may be due to their increased surface area by formation of carbon nanoparticles and of oxygenated functionalities [25, 106] (phenolic, carbonyl and carboxyl [105], quinone [21], or graphite oxide films [2]) during the oxidation of the graphite layer of the PGE accomplished also by an oxidative surface cleaning during the treatment [53, 100]. The oxygen containing groups increase the hydrophilic characteristics of the ETPGE surface [193] and mediate the electron transfer processes [2].

The electrochemical oxidation of aminopurines [43], xanthine, and adenine [194] and of xanthine and its N-methyl-derivatives [195] in the absence and presence of copper ions was investigated by CV, LSV, eliminatory voltammetry with linear scan, DPV, and chronopotentiometric stripping analysis on ETPGE. Similarly, it was shown that using the same working electrode 6-benzylaminopurine (BAP) exhibited, like other aminopurines, an increased oxidation signal in the presence of copper ions. From the electrochemical data a possible stoichiometry and the electrode reaction mechanism of the BPA-Cu complex were established [55]. Other examples of ETPGEs applications along with their analytical performances reported in the literature are listed in Table 2.

#### 2.3.2. PGE Surface Modification

Electrode surface modification is performed in order to enhance the sensitivity and selectivity of the voltammetric determination; for example, Kawde and Aziz reported the improved electrocatalytic activity of a porous Cu-modified PGE in comparison to bare PGE, with respect to the redox behavior of 4-nitrophenol leading to a three-order magnitude lower amperometric detection limit [165].

The PGE active surface can be modified with nanomaterials, polymers, a combination of both, or using other type of modifiers.

There are many different ways to change the surface characteristics of an electrode. Usually one or more compounds (modifiers) are attached to the electrode surface by coating, casting, or dispersion within a conductive matrix in order to combine the chemical/electrochemical/optical or catalytic properties of the modifier(s) with those of the electrode, generating a modified electrode with enhanced figures of merit. A fluorine doped PGE applied to Cr(VI) detection in tap water and smoker urine was obtained by CV in HF solution when fluorinated graphite structures were formed. In SWV the electrode presented two linear ranges, namely, 0.051–0.45 mg·L^−1^ and 0.05–0.4 *μ*g·L^−1^, and a LD of 0.008 *μ*g·L^−1^ Cr(VI) [196], whereas a PGE modified by adsorption of quercetin [192] or hematoxylin [197] exhibited improved electrocatalytic response for sulfide with linear ranges of 1–20 *μ*M and 20–800 *μ*M and a LD of 0.3 *μ*M and 1–200 *μ*M and 0.4 *μ*M sulfide, respectively. An ETPGE modified by adsorption of quercetin was sensitive to reduced *β*-nicotinamide adenine dinucleotide (NADH) in the range 0.5–100 *μ*M with a LD of 0.15 *μ*M. Based on CV data an ECE mechanism was proposed for the electrocatalytic oxidation of NADH [193].

Cibacron Blue (CB) F3GA modified PGE obtained by both CV and passive adsorption was developed to investigate the CB dye affinity to serum albumin. Using CV, the CB current peak decreased linearly with increased bovine serum albumin concentrations in the range 0.25–2.5 mg·mL^−1^ and the obtained LD was 0.15 mg·mL^−1^ [198].

Different metals or metal containing compounds can be attached to bare or already modified PGEs surface either as thin films [28, 64] or as different nanostructures (NS). For example, in situ generated Hg-film PGEs were used for the potentiometric stripping analysis of Cu(II), Pb(II), Cd(II), and Zn(II) from river sediments with comparable results with those obtained by atomic absorption spectrometry [70] or for anodic stripping voltammetric monitoring of Cu(II) levels in the Prut-river during a period of three years [199].

Due to their large surface area, their excellent conductivity, and electrocatalytic properties, nanomaterials enhance the electrodes sensitivity (by high signal-to-background ratio) [33] (e.g., a Pd-nanoparticles modified PGE was able to detect H_2_O_2_ with a LD of 45 nM compared to the LD of 0.58 mM H_2_O_2_ obtained at a bare PGE under the same experimental conditions [24]) and selectivity by improving peak separation [14].

The methodologies for PGE modification with nanomaterials (precious metals nanoparticles (NPs), metal oxide NPs, metal complex nanostructures (NS), and carbon nanotubes) and nanomaterials-conducting polymers composites and the electrochemical applications of such modified PGEs were recently reviewed [33].

Galvanostatic double-pulse technique [200] and CV [201] were applied to obtain NS multilayers thin Cu films and Cu particles modified PGEs, respectively, which exhibited electrocatalytic activity towards nitrate reduction. Copper hexacyanoferrate modified PGE for L-cysteine detection was obtained by cooper electrodeposition on PGE and subsequent immersion in a K_3_[Fe(CN)_6_] solution [132]. PGE modified with MWCNTs (multiwall carbon nanotubes) and Bi-NS obtained by microwave-assisted polyol method were used for Pb(II) determination with a LD of 1.7 nM [202]. A sensitive composite sensor for hydrazine was developed by CV deposition of Cu-NS on MWCNTs modified PGE [149].

Nanoparticles (NP) immobilized on electrodes surfaces improve the electrodes electrochemical characteristics by mediating the electron transfer reactions of electroactive species. NP-modified electrodes can be obtained by electrodeposition [140, 161, 203] or by simply immersion of the pencil lead into the NP solution [25, 32, 188]. Tl_2_O_3_-NS obtained by a sonochemical method were used to develop a Tl_2_O_3_-NS/MWCNTs modified PGE for Cu^2+^ trace sensing with a LD of 4.63 nM Cu^2+^. The developed modified PGE was able to detect three oxidation states of copper [204].

Carbon nanomaterials, as carbon nanotubes (CNTs) [120, 135, 158, 204, 205], nanofibers [206], nanopowders [68], or graphene [28, 147, 175, 177], are largely used for the development of electrochemical sensors. CNTs are NS very suitable for PGEs surface modification due to their nanometer dimensions, high stability, large specific area, good conductivity, electronic properties, and topological defects conferring them excellent catalytic characteristics which enable the promotion of redox reactions at the electrode surface (improvement of electron transfer rate and redox reversibility), and the ability to reduce the electrode surface fouling and the electrolyte impedance [3, 120, 158, 161, 205].

Particularly selectivity but also the sensitivity of the voltammetric determinations can be improved by covering the electrode (PGE) surface with a polymer bearing chemical groups able to interact differently with various compounds or with molecularly imprinted polymers (MIP) whose interaction with diverse species is restricted by both its functional groups and the shape and size of the cavity resulted after template removal from the polymer.

Recently, polymer modified electrodes gained great interest due to their good stability, reproducibility, more active sites, homogeneity in electrochemical deposition [138], and improved response characteristics [156]. One of the most used technique for covering the electrode surfaces with polymeric films is electropolymerization; it can be realized by cyclic voltammetry [34, 59, 66, 114, 118, 121, 122, 130, 138, 156, 168, 170, 173, 175, 207, 208], by applying a constant potential [41, 112, 183] or a constant current [62, 153, 172, 178] to the bare or previously modified PGE immersed in the solution containing monomer and eventually other components. A dip-coating method was applied to modify a PGE from a casting solution containing carmoisine/polyaniline or copper-carmoisine complex/polyaniline [129]. To enhance the polymers ability to recognize and bind selective the analyte, they can be molecularly imprinted with the target/analyte molecule (template) by chemical grafting, soft lithography, molecular self-assembly, electropolymerization (galvanostatic, potentiostatic, and cyclic voltammetric methods), irradiation, or heating [118, 168, 169, 173], followed by the template extraction from this polymer resulting a tailor made receptor that can recognize and rebind the analyte according to its shape and functionality. Besides their increased selectivity, molecularly imprinted polymers- (MIP-) modified PGEs have also other advantages like ease of preparation and low-cost and high chemical stability under various circumstances [111, 125–127, 148, 161, 175], but they also have some possible drawbacks as incomplete template removal, embedding of binding sites, analyte constraint accessibility to the template cavities, and electroinsulating [111, 141, 175]. Hybrid nanocomposites combining the advantages of their constituents (e.g., MIP and nanomaterials) have been used for electrode surface modification in order to overcome the above-mentioned disadvantages of MIPs and to improve the electrochemical and sensing characteristics of the PGE through a synergistic effect. Therefore, the chemical stable polymer acts as a “recognition element” which reduces interferences and responds quickly by selective recognition of the analyte molecules via ion exchange and electrostatic and/or hydrophilic/hydrophobic interactions whereas the nanomaterials, due to their unique and specific electroanalytical characteristics, increase the conductivity and improve the analyte mass transport and charge transfer, enhancing the electrochemical activity towards the compound of interest [14, 33, 68]. On the other hand, molecular imprinting on nanomaterials, after template removal, will improve the analyte targeted accessibility to the electron transfer site at the electrode surface reducing the mass transfer resistance [148]. Rezaei et al. [61] explain the role of each component in the stepwise development of modified Au nanoparticles-imprinted sol-gel-MWCNTs-PGE sensitive to ranitidine.

Hybrid nanocomposite PGEs have a high surface to volume ratio, more functional groups that constitute specific binding sites, higher adsorption capacity, excellent recognition ability, and thus very good sensitivity and selectivity, but also good reproducibility and stability due to the three-dimensional nanocomposite MIP structure. So a nanocomposite MIP-Au-NPs-PGE used for sensitive (LD 0.9 nM) and selective caffeine determination from real samples without interference of compounds with similar structure displayed good construction reproducibility (RSD 4.4%), repeatability (RSD 3.7%), and stability of over two weeks [60]. The immobilization of a MIP onto a functionalized MWCNTs modified PGE resulted in a very sensitive (LD 0.28 ng·mL^−1^) electrode for *γ*-aminobutyric acid detection at ultra-trace levels in a wide concentration range (0.75–205.19 ng·mL^−1^) from biological samples without any cross-reactivity and false positives. The electrode presented good preparation reproducibility (RSD 3.3%), regeneration ability, and stability (one month) [111]. A MI-polyaniline ferrocene-sulfonic acid-carbon dots PGE could be used for over 40 days (when stored at room temperature) for chiral sensitive (LD at 10^−12^ M level), accurate (RSD 3.4%), and selective recognition of L- and D-ascorbic acid from biological fluids in the presence of structurally close chiral or nonchiral molecules [115]. It is worth mentioning that the modified electrode surface was easy regenerated either by analyte elution using an adequate electrolyte [60] or by electrochemical dedoping [115].

Materials of the MWCNTs-MIP-template adduct type were usually obtained by molecular imprinting on nanostructures and can be easily covered onto PGEs [61, 65, 67, 111, 116, 117, 136, 148, 150, 159–161]. Applications and performance characteristics of the above-mentioned and also other modified PGEs are listed in Table 3.

Stability and reproducibility are important parameters for the electrode performance; the modification can improve the properties of substrates, the stability, and reproducibility of the electrode response.

The tested room temperature and long-term stabilities of modified PGEs from several days to more than 100 days showed good stability. Thus, ETPG electrodes conserved their activity for a long time (i.e., 30 days) when they are stored in a vacuum desiccator [21, 57, 106]. The results obtained for the working stability of some modified PGEs investigated by monitoring the analyte peak after more consecutive runs on a single modified electrode indicated a decrease of the peak current less than 5%. A single electrode could be used after regeneration for 30 [25, 116] to 90–100 consecutive runs [66, 67, 159] with quantitative recoveries. The response of some modified electrodes, especially modified with conducting polymers evaluated for a long period of time, showed that the sensor kept 98% of the initial performance after 70 days and 81% even after 140 days [34] or 96% of its initial response after 105 days [169].

The good reproducibility of the modified PGEs is correlated with the electrode stability. The fabrication reproducibility of more than five different electrodes, prepared under the same conditions, showed acceptable reproducibility with a RSD value between 0.95% and 4.9% [21, 57, 60, 62, 67, 106, 117, 125, 133, 160, 175]. Similar results for the reproducibility were obtained in the case of the insulin determination with a RuO_2_-graphene oxide PGE [188], the phenol detection using a precharged disposable PGE [104], and the sulfide quantitation with a PGE modified with hematoxylin [197], namely, RSD% values of 3.7, 3.76, and 4.1%, respectively. Repetitive measurements carried out for 10 successive runs showed a RSD between 0.87% and 1.36% [65–67, 116, 117, 204]. So the reproducible current response with a single electrode proves the stability of the electrode.

#### 2.3.3. Other Applications

A microfluidic chip containing an N-doped carbon nanofiber modified PGE as working electrode was recently described for the simple electrochemical detection of hydroxyl radicals in cigarette smoke with a detection limit of 10^−6^ M [206]. A PANI-film modified PGE acting as a pH sensor was connected between the tips of the working and counter electrodes coupled to a potentiostat generating thus an amperometric pH-controllable molecular switch that forms a measuring system together with a suited reference electrode immersed in the analysed solution [207, 208].

Due to a complex matrix of the sample to be analysed or in order to improve the selectivity and sensitivity, the electrochemical detection methods are often coupled to separation methods. Amperometric oxidation of phenols and chlorophenols at disposable GRC was used as detection mean in liquid chromatography [209]. Ensafi et al. [187] reported the use of a PGE modified with a thiol-functionalized silica film incorporating SDS (sodium dodecyl sulphate) which extracts and preconcentrates the reduced form of flutamide due to the applied potential. Subsequently, the accumulated analyte was stripped from the electrode surface and determined by DPV. Insulin was preconcentrated by extraction into a SDS supported liquid membrane by formation of a SDS-insulin complex which was further reextracted into a small volume of acceptor phase and was analysed by DPV on a modified PGE [188]. Other examples of PGEs used in combination with separation techniques are presented in Table 4.

PGEs modified by electrodeposition of tartrate-doped polypyrrole [210], different aniline [211–213], or thiophene [214–216] derivatives based polymers were investigated in order to obtain electrode active materials for supercapacitors application. In recent years, graphite pencils were used for the development of interesting electronic applications like bilayer chemiresistor [217], photoconductive PbS photodetector realized by pencil graphite electrodes drawn on stone paper acting as substrate, or a hand drawn pencil electrode for determination of lead traces in water [73], dopamine [74], or p-nitrophenol [30]. It was demonstrated that pencil drawn electrodes present the best reversibility and the highest signal if they were drawn 10 times with a 6B pencil [75]. Doped pencil leads for paper based analytical devices with chemical modified electrodes have been also reported [76]. Pencil leads doped with Ag and AgCl were used to draw carbon-based Ag/AgCl reference electrodes [77]. A paper based potentiometric electronic tongue comprising three pencil drawn sensors sensitive to Cl^−^, Na^+^/K^+^, and Ca^2+^/Mg^2+^ was able to make the difference between different types of mineral water and between tap and mineral water [78]. Pencil-on-paper devices can be employed as analytical tools [79–81] or as electronic devices [82–85].

### 2.4. Techniques Used for Surface Characterization of Bare or Modified PGEs

In order to demonstrate the modification of a surface, different techniques are used to investigate and to compare the morphology and/or the electrochemical characteristics of the respective surface and/or of the modifier, before, during, or after the modification process. For surface morphology of bare or modified PGE investigations, different techniques of electron [3, 15, 24, 28, 29, 32, 34, 35, 54, 60, 65–67, 85, 111, 118–122, 126–128, 130, 135, 141–143, 145–148, 158, 161, 170, 173, 175, 178, 181–183, 189, 198, 200, 204, 205, 210, 216, 218, 219] and atomic force microscopy [28, 29, 60, 65–67, 112, 126, 172, 198, 205] are widely used. Sometimes, scanning electron microscopy (SEM) was coupled with energy-dispersive X-ray spectroscopy analysis [130, 143, 149, 152, 156, 172, 187, 188, 203–205]. X-ray diffraction was applied to investigate the chemical composition and/or crystal structure [143, 145, 203, 204, 206]. Fourier transform-infrared (FT-IR) investigations were performed to emphasize the presence of certain functional groups indicating the chemical structure modification during the chemical reactions, like polymerization [3, 29, 65, 117, 121, 122, 128, 130, 141, 143, 146, 148, 156, 170, 175, 189, 205, 210]. FT-IR and Raman spectroscopy were also applied to determine the structure modification from graphite to graphene oxide [28, 147, 194].

Electrochemical impedance spectroscopy (EIS) provided information on impedance changes at the electrode surface and therefore on the appearance of surface modifications enabling thus the differentiation between unmodified and modified electrodes [3, 54, 60, 61, 104, 119, 127, 140, 149, 161, 170, 173, 182, 198, 205, 210, 216], while CV is useful to characterize an electrode from electrochemical point of view or to investigate the electroactivity of conducting polymers, providing information about the formation, the potential, and the anodic/cathodic peak potentials of the polymer [3, 15, 24, 28, 32, 43, 54, 60–63, 66, 67, 72, 104, 111, 114, 117, 118, 120–122, 125, 127, 135, 140, 144, 145, 149, 152, 158, 161, 168, 170, 175, 177, 181, 183, 192, 193, 203–205, 208, 210].

Some examples of these techniques used in the development of modified PGEs are listed below. Thus, SEM images of bare 2B-type PGE showed a smooth surface [29, 140] and for H-type pencil leads they revealed a porous, dense surface due to the homogeneous dispersion of graphite powder in the organic binder [158], while for the modified PGE they indicate a homogeneous distribution of the MWCNTs on the electrode surface [158], spindle-like Au-nanostructures [140], or different structures for sol-gel modified PGE, MIP-folic acid layer and MIP-layer on the sol-gel modified PGE [29]. SEM investigations revealed that the density of copper nanostructures on PGE is higher than on GCE or pyrolytic graphite electrodes and this fact is due to the PGE higher active surface area [201].

Metals electrodeposition is of high technological importance, for example, in the electronic industry as coatings. Thus, the mechanism of copper electrodeposition on PGE was investigated by CV and chronoamperometry, while SEM was used to observe the morphology of copper nuclei obtained in different experimental conditions [218]. Pd electrocrystallization mechanism on PGE was established based on the current transient curves. SEM micrographs indicate a homogeneous distribution of hemispherical Pd clusters on the PGE [219].

Raman spectroscopic examinations of the PGE carbon structure and SEM micrographs of the PGE surface before and after DPV oxidation of acyclovir emphasized no modifications during acyclovir electrochemical measurement, confirming thereby the stability of the PGE surface [27]. In the case of the Cu-microparticles modified PGE, SEM analysis showed that the surface morphology of the Cu-microparticles changed during the electroanalysis of valacyclovir due to the adsorption of the Cu-valacyclovir complex [180].

## 3. Conclusions

The data included in this paper were compiled after carefully studying, comparing, and correlating the information found in over two hundred articles that appeared in the last two and a half decades approaching this topic. The review pointed out the most important features of PGE as working electrode in comparison to other graphite based electrodes, emphasizing its even better electrochemical performances in some cases, but most of all its simplicity and high cost-effectiveness ratio. Due to the graphite pencil leads widespread availability (they can be purchased in local market at low costs) they constitute an important single-use electrode material. Therefore, PGEs are cheap and easy disposable, having also an adjustable area of the working surface, enabling shorter analysis times, and offering the possibility of determining low concentrations in small sample volumes. PGEs can be also readily miniaturized or modified. Thus, they can be employed as working electrodes as such or by modifying their surface either by electrochemical pretreatment or by covering it with different types of materials, ranging from simple compounds to various imprinted or nonimprinted polymers, nanomaterials, or combinations of these (nanocomposites), leading to improved selectivities and sensitivities. The techniques used to characterize the bare or modified PGEs are also listed together with some practical examples. Electroanalysis using PGEs include potentiometric, amperometric, or different voltammetric techniques and proved to reach wide linear ranges and low detection limits. All these resulted in the widespread applicability of PGEs to the determination of different inorganic and organic species from environmental, archeological, food, pharmaceutical, and clinical samples. It is worth mentioning the development, in recent years, of flexible paper based analytical electrochemical devices which use pencil drawn electrodes. Consequently, pencil graphite electrodes have proven to be a viable and economical electroanalytical tool.

Besides the various analytical applications, graphite pencils also found interesting uses in electronics.

## Figures and Tables

**Table 1:** Electroanalytical applications of unmodified PGEs.

Compound	Technique	Linear range (*μ*M)	LD (*μ*M)	Sample	Ref.
Acebutolol	DPV SWV	1–15	0.0126 0.0128	Urine	[90]
Acetaminophen	CV; FI	100–5000		Pharmaceuticals	[40]
Acetylsalicylic acid	SWV	35–300^*∗∗*^ 350–700	167^*∗*^	Pharmaceuticals	[91]
Acyclovir	DPV	1–100	0.3	Pharmaceuticals	[27]
Caffeic acid/TPC	FIA-Amp	1–20^*∗∗*^		Tea infusion	[19]
Caffeic acid/TPC	DPV	0.1–3000	0.0883	Tea infusion	[10]
Caffeine	SWASV	<500^*∗∗*^	9.2^*∗∗*^	Tea	[92]
Chlorogenic acid	DPV	0.1–500	0.0714	Dietary supplements	[46]
Dantrolene sodium	DPV SWV	0.395–2.955^*∗∗*^ 0.395–1.9^*∗∗*^	0.09 0.052	Pharmaceuticals, human mother milk, urine	[48]
Eugenol	DPV	0.3–50	0.085	Pharmaceuticals	[36]
Famotidine	DPV	0.472–495	0.151	Pharmaceuticals	[45]
Flutamide Irinotecan	SWCASV	0.398–6.36 0.0794–0.403	0.0155 0.00168	Bulk form, human urine, serum	[49]
Hydroxyurea	DPV	10–1000	7.89	Pharmaceuticals, human urine	[31]
Itraconazole	SWASV	10.6–127^*∗*^	8.7^*∗*^	Pharmaceuticals, biological fluids	[93]
Methylergometrine maleate	DPV	0.10–1.00^*∗∗*^	0.02^*∗∗*^	Pharmaceuticals	[52]
Niclosamide	DPV	0.05–10.0	0.015	Pharmaceuticals	[37]
Ozone	Amp	30–400	0.6	Tap water	[38]
Paclitaxel	DPV	0.4–3.0	0.00246	Pharmaceuticals, biological fluids	[26]
Rosmarinic acid	DPV	0.01–10	0.00793	Tea infusion	[11]
Salicylic acid	SWV	0.1-80^*∗∗*^	1.7^*∗*^	Willow barks and branches	[94]
Silver	DPV	0.015–0.25	0.01	Waters, tobacco cells, fish tissues	[95]
Strontium ranelate	SWV	1–10^*∗∗*^	0.17^*∗∗*^	Pharmaceuticals	[20]
Trepibutone	SWV	0.24–10^*∗∗*^	20^*∗*^	Pharmaceuticals	[96]
Uranium	SWASV	<0.00008^*∗*^	0.000007^*∗*^	Rocks, earthworm	[97]
Vitamin B1 Vitamin B6	DPV	10.0–1000 5.00–1000	5.34 2.81	Pharmaceuticals	[44]

^*∗*^ng·mL^−1^; ^*∗∗*^*µ*g·mL^−1^; DPV: differential pulse voltammetry; SWV: square wave voltammetry; CV: cyclic voltammetry; FI(A): flow injection (analysis); Amp: amperometry; SWASV: square wave anodic stripping voltammetry; SWCASV: square wave cathodic adsorptive stripping voltammetry; TPC: total polyphenolic content.

**Table 2:** Electroanalytical applications of ETPGEs.

Compound	Technique	Linear range (*μ*M)	LD (*μ*M)	Sample	Ref.
Alkylphenols	DPV			Water	[50]
4-Nonylphenol		1.20–94	0.420		
4-Octylphenol		0.6–78	0.250		
4-tert-Octylphenol		2.38–243	0.770		

4-Aminophenazone Caffeine	DPV	1–11 1–9	0.0796 0.00981	Real samples	[98]

Benzo(a)pyrene	SWSV	0.25–1.25	0.027	Spiked human urine	[99]

Bisphenol A	AdDPSV	0.05–5; 5–10	0.0031	Tape and river water	[54]

Boron	DPV	280–12000^*∗*^	84^*∗*^	Water, steel	[4]

Capsaicin, dihydrocapsaicin	AdSWSV	0.016–0.32 0.0033–0.33	0.0037 0.00091	Turkish pepper	[25]

Chlorpromazine	DPV	0.35–35^*∗*^	0.2^*∗*^	Human plasma, urine	[22]

Cinchocaine hydrochloride	DPV: ox red SWV: ox red	1.5–18 1.3–13.6 1–11 1.3–17.7	0.664 0.458 0.452 0.483	Spiked human urine	[58]

Dopamine	ATSDPV	0.01–5.0	0.00031	Blood serum	[21]

Dopamine Uric acid	DPV	0.15–15 0.3–150	0.033 0.12	Pharmaceuticals, body fluids	[2]

Gluten	DPV	20–100^*∗∗*^	7.11^*∗∗*^	Flour, vinegar, baker's yeast	[56]

Indole-3-acetic acid Kinetin^a^	SWV	0.5–2; 2.5–10	0.14 0.11	Maize plant extracts	[100]

Methamphetamine	DPV	0.074–54	0.05	Human serum, urine	[101]

Morphine	DPV, Amp	1–100	0.26	Human urine, street drug samples	[102]

Nalbuphine hydrochloride	DPV SWV	16–150	6.38 11.3	Spiked urine, pharmaceuticals	[103]

*α*-Naphthol	LSASV	0.01–2.0	0.0015	Water	[47]

Nicotine	SWV	7.6–107.5	2.0	Cigarettes	[53]

Paracetamol	ATSDPV	0.05–2.5	0.0025	Blood serum	[57]

Phenol	SWV	0.05–1	0.00417	Water	[104]

Phenol, m-cresol	DPV	40–320	0.120	Insulin formulations	[9]

Quercetin	DPASV	0.001–1.5	0.0003	Cranberry, blackcurrant juices	[105]

Tannic acid	DPASV	0.005–0.5	0.0015	Beverages	[51]

Uric acid	DPV	0.05–10	0.0015	Body fluids	[106]

Uric acid	DPV	1–160	0.19	Human serum, urine	[107]

Vitamin C	Amp	1–100	1.18	Human plasma	[69]

^a^In solution: SDS (sodium dodecyl sulphate) < critical micellar concentration; ^*∗*^ng·mL^−1^; ^*∗∗*^*µ*g·mL^−1^; SWSV: square wave stripping voltammetry; AdDPSV: adsorptive differential pulse stripping voltammetry; AdSWSV: adsorptive square wave stripping voltammetry; ox: oxidation; red: reduction; ATSDPV: adsorptive transfer stripping voltammetry differential pulse voltammetry; LSASV: linear sweep anodic stripping voltammetry; DPASV: differential pulse anodic striping voltammetry. All the other abbreviations are like those in Table 1.

**Table 3:** Electroanalytical applications of modified PGE.

Compound	Modifier	Technique	Linear range (*μ*M)	LD (*μ*M)	Sample	Ref.
Acetaminophen	Poly[2,5-di(2-hiophenyl) -1-p-(tolyl)Pyrrole]	DPV	0.025–5	0.00194	Pharmaceuticals	[108]

Acyclovir	ET *β*-cyclodextrin polymer	SWAdSV	0.05–0.6 1–9	0.00759	Dosage forms, urine pharmacokinetic studies	[109]

2-Amino-benzimidazole	MI-PPy	DPV	0.001–30000	0.0001	Biological model samples	[110]

*γ*- Aminobutyric acid	MI-FUAA/MWCNTs-COOH	DPASV	0.75–205.19^*∗*^	0.28^*∗*^	Cerebrospinal fluid, human serum, aqueous	[111]

Arsenic(V)	PPy-AsO_4_^3−^	Pot	50–100000	28	Water	[112]

Arsenic(III)	Au-NDs/CNTs	ASV	0.5–80^*∗*^	0.4	Tap water	[113]

Ascorbic acid	MI-PPy	DPV	250–7000	74	Pharmaceuticals	[114]

D-, L- Ascorbic acid	MI-PANI-FSA-C-dots	DPV	0.006–0.155 0.006–0.165	0.000002 0.000001	Pharmaceuticals, human plasma	[115]

D-, L- Aspartic acid	MI-PI3AA/MWCNTs	DPASV	0.15–7.4 0.15–8.64	0.025 0.016	Pharmaceuticals, blood serum	[116]

D-, L- Aspartic acid	MI-NAPD/MWCNTs-TiO_2_-NPs-CHPA	DPASV	9.98–516.25^*∗*^ 9.98–532.72^*∗*^	1.71^*∗*^ 1.76^*∗*^	Cerebrospinal fluid, blood serum, pharmaceuticals	[117]

Benzimidazole	MI-PPy	DPV	3–5000	0.7	Biological	[118]

Bisphenol A	Au-NPs/PVP	SWAdSV	0.03–1.1	0.001	Bottled drinking water	[119]

Bisphenol A	PANI-NRs/MWCNTs	Amp	1–400	0.01	Spiked boiling water	[120]

Cadmium	P4VP	Pot	0.1–100000	0.0251	Environmental, food, biological	[121]

Cadmium	Poly(4VP-co-Ani)	Pot	1–10000	0.794	Sea water, tomato, rice, ayurvedic medicine	[122]

Caffeine	Au-NPs/MIP-sol-gel	SWV	0.002–0.05 0.05–1.0	0.0009	Urine, plasma, tablets, tea, energy and soda drink	[60]

Carminic acid	Pd-Au-NPs/PPr	SWV	0.01–1	0.0059	Popping candy	[123]

Ceftriaxone sodium	Copper hexacyanoferrate	CV, chrono-amp	2–72	0.54	Pharmaceuticals	[124]

Ciprofloxacin	MI-PPy and o-PD	SWV	0.001–1000	0.0000758	Tablets	[125]

Chlorogenic acid	MI-PPy	Pot	1–10000	1	Coffee	[126]

Chlorpyrifos	MI-PPy	EIS	20–300^*∗*^	4.5^*∗*^	Soil	[127]

Copper	Alga-OMNiIIP	DPASV	0.008–7.807^*∗*^	0.0018^*∗*^	Environmental, pharmaceuticals, blood serum	[128]

Copper	Cu-carmoisine/PANI	Pot	5–100000	2	Tea leaves, black currants, sour cherry juice	[129]

Copper	PPy-SSA	Pot	10–100000	5.42	Electrical copper wire	[130]

Copper	PPy-P4R	Pot	10–50000	6.7	Electrical copper wire, tap water	[131]

Copper	PSS–CnP	DPASV	<1000^*∗*^	0.00173	Mineral, river and sea water	[68]

L-Cysteine	Copper hexa-cyanoferrate NS	chrono-amp	1–13	0.13	Urine	[132]

Dextromethorphan	Poly(diallyl-dimethyl-ammonium chloride)/MWCNT/CQDs	DPV	2–600	0.2	Cough syrups, human urine, plasma	[133]

Diazepam	ET Bi	DPV	1.4–16.7	1.1	Tablets, urine	[134]

Diclofenac sodium	MWCNTs	DPV	0.047–12.95	0.017	Biological samples, pharmaceuticals	[135]

1,4-Dihydroxy-anthraquinone	MI-PPy/MWCNTs-COOH	DPV	0.01–100	0.00415	Serum, plasma	[136]

Dipyrone	PPy-dipyrone	Pot	100–40000	86.2	Spiked human urine, Pharmaceuticals	[62]

Dopamine	Poly(xylenol orange)	DPV	2–9	0.091	Injections	[137]

Dopamine Uric acid	Poly(amaranth)	DPV	0–250 0–600	0.05 0.2	Injections	[138]

Dopamine Uric acid	Poly(Naphthol Green B)	DPV	—	—	Injections	[139]

L-Dopa	Au	DPV	20–100	1.54	*Mucuna pruriens*	[140]

Epinephrine	MI-poly(NHPM-MWCNTs)	DPASV	0.09–5.90^*∗*^	0.02^*∗*^	Pharmaceuticals, blood serum	[141]

Estradiol	Au-NPs/MWCNTs	LSV	0.07–42	0.01	Blood serum	[142]

Ferritin	Imprinted Ag@CdS	DPV	1.99–23.43^*∗∗*^	0.65^*∗∗*^	Blood serum	[143]

Fluoxetine	PVC/PEDOT-C14	ITSV	0.1–1.0	0.035	Tap and river water	[144]
Citalopram				0.025		
Sertraline				0.045		

Folic acid	MI-pTAT/sol-gel	DPCSV	0.007–0.156^*∗∗*^	0.0021^*∗∗*^	Blood serum, pharmaceuticals	[29]

Glucose	OxPPyNFs-CoPcTS	DPV	250–20000	100	Serum samples	[34]

Glucose	Cu-NPs	Amp	1–100 100–100000	0.44	Blood serum	[145]

Glyphosate	MI-pMAC/AuNPs	DPASV	3.98–176.23^*∗*^	0.35^*∗*^	Aqueous, soil, human serum	[146]
Glufosinate			0.54–3.96^*∗*^	0.19^*∗*^		

Heavy metals	Bi-film	SWASV	0.01–0.12	Pb: 0.0024 Cd: 0.0029 Zn: 0.012	Simultaneous in situ, aqueous solutions	[64]

Heavy metals	Nafion-graphene nanocomposite-Bi-film	SWASV	2–20^*∗∗*^ 10–100^*∗∗*^	Pb: 0.13^*∗∗*^ Cd: 0.09^*∗∗*^ Zn: 0.17^*∗∗*^	Tap water	[147]

Heavy metals	ERGO-Bi-film	SWASV	2–100^*∗*^	Pb: 0.12^*∗*^ Cd: 0.09^*∗*^ Zn: 0.19^*∗*^	Tap water	[28]

Hemoglobin	MI-pNMGA/QDs-MWCNTs	DPCSV	27.8–444.0^*∗*^	6.73^*∗*^	Blood	[148]

L-Histidine	CIP/MWCNTs	DPASV	9.990–323.572^*∗*^	1.980^*∗*^	Pharmaceuticals, blood serum	[67]

Hydrazine	Au-NPs	SWV Amp	0.05–1000 25–1000	0.042 3.07	Drinking water	[32]

Hydrazine	Cu-NS/MWCNT	SWV Amp	0.1–800 50–800	0.07 4.3	River, waste, well waters	[149]

Hydrochlorothiazide	MI-PPy/MWCNTs-COOH	DPV	0.0009–10 10–10000	0.0001	Pharmaceuticals, serum	[150]

Hydrogen peroxide	Ag/FeOOH nanocomposites	Amp	30–15000	22.8	Disinfectant	[151]

Hydrogen peroxide	Pd-NPs	Amp	10–140	0.045	—	[24]

Hydrogen peroxide	Pt-NPs	Amp	10–110	3.6	—	[152]

Insulin	MI-pAEP-MWCNTs	DPASV	0.000058–0.005647	0.0000186	Pharmaceuticals, blood serum	[65]

Iodide	PPy-iodide	Pot	10–100000	9.3	Pharmaceuticals	[153]

K^+^, Na^+^	PB	CV	1000–500000	600–12000 600–21000	Synthetic	[63]

Lorazepam	MI-PPy/sol-gel/AuNPs	SWV	0.0002–0.002 0.002–0.020	0.00009	Pharmaceuticals, plasma, urine	[154]

Lomustine	MF	SWCASV	0.192–13.6	0.0813	Human blood, urine	[155]

Magnesium	PPy-Titan yellow	Pot	10–50000	6.28	Tap and mineral water	[156]

Mancozeb	SPIONs/MISP	—	5.96–257.0^*∗∗*^	0.96^*∗∗*^	Soil, vegetable	[157]

Methadone	MWCNTs	DPV	0.1–15	0.087	Human serum, urine	[158]

D-, L- Methionine	MI-polyBz/MWCNTs-COOH	DPV	11.7–188.7^*∗*^ 11.7–206.3^*∗*^	3^*∗*^ 3^*∗*^	Pharmaceuticals, blood serum	[159]

Metoprolol	MI-PPy/MWCNTs-COOH	DPV	0.06–490	0.00288	Pharmaceuticals, serum	[160]

Morphine	Au-NPs/MIP/MWCNT	SWV	0.008–5	0.0029	Human urine, serum	[161]

Nitrate	PPy-NO_3_^−^	Pot	100–100000	50	Water	[162]

Nitrite	Polypyronin Y	Amp	1–100	0.5	Food	[163]

Nitrite	PVF+/MWCNTs	DPV	1–400	0.1	Mineral water	[164]

4-Nitrophenol	MWCNTs	FI-Amp	5–300^*∗*^	1.5^*∗*^	Water	[35]

4-Nitrophenol	Cu	Amp	50–850	1.91		[165]

2-Nitrophenol 4-Nitrophenol	Bi	DPV	2–200 4–400	5.42 7.48		[166]

Pantoprazole	PPy-PTZ	Pot	10–11000	6.9	Pharmaceuticals	[167]

Paracetamol	MI-PPy	DPV	5–500 1250–4500	0.79	Pharmaceuticals	[168]

Phenothiazine	MI-PPy	DPV	1–300 500–10000	0.3	Model and real biological	[169]

D-, L- Pyroglutamic acid	MI-poly[5-MTCA–Cu(II)]	DPASV	2.8–170.0^*∗*^	0.77^*∗*^	Cerebrospinal fluid, blood plasma, urine	[66]

Quercetin	p-AMT	DPV	0.1–6^*∗∗*^	2.2	—	[170]

Ranitidine	Au-NP/MIP-sol–gel/MWCNTs-COOH	SWV	0.05–2.0	0.02	Human urine	[61]

Serotonin	p(P3CA)	AdDPSV	0.01–1.0	0.0025	Blood serum, urine	[171]

Sildenafil citrate	PPy-citrate	Pot	34–1700	30	Pharmaceuticals	[172]

Sulfanilamide	MI-PPy	DPV	0.05–1.1 1.1–48	0.02	Spiked human serum, ground water	[173]

Sulfamethoxazole	MI-OxPPy	DPV	25–750	0.359	Pharmaceuticals	[59]

Thiocarbohydrazide	MI-PPy	DPV	500–6000	50	Biological	[174]

6-Thioguanine	MI-polyneutral red/ERGO	DPASV	0.124–78.0^*∗*^	0.02^*∗*^	Pharmaceuticals, urine, serum	[175]

Triamterene	MI-PPy/MWCNTs-COOH	DPV	0.08–265	0.00335	Serum, pharmaceuticals	[176]

L-Tyrosine	graphene	SWV	0.8–60	0.07	Urine	[177]

Uranium(VI)	PPy-ZnUO_2_(CH_3_COO)_4_	Pot	1–10000	0.63	Mineral and tap water	[178]

Uric acid	Ox-PEDOT-nf	DPV	0.01–20.0	0.0013	Blood serum, urine	[179]

Valacyclovir	CuMPs	AdSWV	0.002–0.01	0.000178	Pharmaceuticals	[180]

Vitamin B12	PNT	SWV	0.2–9.5	0.093	Tablets	[181]

Vitamin B12	SWCNTs-chitosan	SWV	0.005–0.1	0.00089	Pharmaceuticals	[182]

Zinc	Tartrazine-PPy	Pot	10–100000	8	Barley flakes, rice dry milk	[183]

^*∗*^ng·mL^−1^; ^*∗∗*^*µ*g·mL^−1^; ET: electrochemically treated; MI: molecularly imprinted; PPy: polypyrrole; FUAA: 5-fluorouracil-N-acetyl acrylamide; NDs: nanodendrites; CNT: carbon nanotubes; ASV: anodic stripping voltammetry; FSA: ferrocene sulfonic acid; PI3AA: poly(indole-3-acetic acid); NAPD: N-acryloyl-pyrrolidine-2,5-dione; NPs: nanoparticles; CHPA: 2-chloro-N-(4-hydroxyphenyl)-acetamide; PVP: polyvinylpyrrolidone; SWAdSV: square wave adsorptive stripping voltammetry; PANI: polyaniline; NRs: nanorodes; MWCNTs: multiwall carbon nanotubes; P4VP: poly(4-vinyl pyridine); poly(4VP-*co*-Ani): poly(4-vinylpyridine-*co*-aniline); MIP: molecularly imprinted polymer; PPr: polyproline; chronoamp: chronoamperometry; o-PD: o-phenylenediamine; EIS: electrochemical impedance spectroscopy; OMNiIIP: one monomer ion imprinted polymer; SSA: sulfosalicylic acid; P4R: Ponceau 4R azo dye; PSS-CnP: polystyrene sulfonate-carbon nanopowders; NS: nanostructure; CQDs: carbon quantum dots; NHPM: N-hydroxyphenyl maleimide; LSV: linear sweep voltammetry; Ag@CdSNPs: AgCdS core shell nanoparticles; PVC: poly(vinyl chloride); PEDOT-C14: 3,4-ethylenedioxythiophene; ITSV: ion transfer stripping voltammetry; pTAT: poly(triaminotriazine); DPCSV: differential pulse cathodic stripping voltammetry; OxPPy: overoxidized polypyrrole; NFs-nanofibers; CoPcTS: cobalt(II) phthalocyanine tetrasulfonate; pMAC: poly(N-methacryloyl-L-cysteine); ERGO: electrochemically reduced graphene oxide; pNMGA: poly(N-methacryloyl glutamic acid); QD: quantum dots; CIP: complex imprinted polymer; pAEP: poly[(p-acryloylaminophenyl)-{(4-aminophenyl)-diethyl ammonium}-ethylphosphate]; PB: Prussian Blue; MF: mercury film; SPIONs: superparamagnetic iron oxide nanoparticles; MISP: molecularly imprinted star polymer; Bz: benzidine; PVF: poly(vinylferrocene); PTZ: pantoprazole; MTCA: methyl-2-thiophene carboxylic acid; p-AMT: polymerized 5-amino-2-mercapto-1,3,4-thiadiazole; p(P3CA): poly(pyrrole-3-carboxylic acid); Ox-PEDOT-nf: overoxidized poly (3,4-ethylene-dioxythiophene) nanofibers; CuMPs: copper micropoarticles; PNTs: peptide nanotubes; copper microparticles; SWCNTs: single-wall carbon nanotubes. All the other abbreviations are like those in the previous tables.

**Table 4:** PGE used in tandem with separation techniques.

Compound	PGE modifier	Technique	Linear range (*μ*M)	LD (*μ*M)	Sample	Ref.
D-, L- Aspartic acid	MIP	MIP-SPME-DPASV	0.6–10^*∗*^ 0.1–10^*∗*^	0.180^*∗*^ 0.032^*∗*^	Blood serum, cerebrospinal fluid	[184]

Buprenorphine	MWCNTs	PTFE-LPME- DPV	0.000001–0.000109 0.000109–0.11	0.0000006	Human urine and plasma	[185]

Clozapine	—	EME-DPV	3–1500^*∗*^	0.9^*∗*^	Human plasma	[71]

Epinephrine	MIP/MWCNTs-NHPM	MI-MSPE-DPASV	0.005–8^*∗*^	0.002^*∗*^	Cerebrospinal fluid, plasma, pharmaceutics	[186]

Flutamide	SDS-modified silica film	DPV	0.0001–0.1 0.1–100	0.000034	Human urine and plasma	[187]

Insulin	RuO_2_-GO nanocomposite	DPV	0.0008–0.02 0.02–1.00	0.000024	Blood, urine	[188]

D-, L- Methionine	EC-MIP/MWCNTs-COOH	MI-MSPE- DPCSV	0.03–30.00^*∗*^	0.0098^*∗*^	Pharmaceuticals, human blood plasma	[189]

Tramadol	RGO	EM-SPME- LSV	0.01–0.50^*∗∗*^ 0.50–50^*∗∗*^	0.003^*∗∗*^	Urine	[190]

^*∗*^ng mL^−1^; ^*∗∗*^*µ*g mL^−1^; MI(P): molecularly imprinted (polymer); SPME: solid phase microextraction; PTFE: polytetrafluorethylene; LPME: liquid three-phase microextraction; EM(E): electromembrane (extraction); MI-MSPE: MI-micro-solid-phase extraction; SDS: sodium dodecyl sulphate; (R)GO; (reduced) graphene oxide; EC-MIP: electrochemically synthesized-MIP. All the other abbreviations are like those in the previous tables.

## References

[1] Chehreh Chelgani S., Rudolph M., Kratzsch R., Sandmann D., Gutzmer J. (2016). A review of graphite beneficiation techniques. *Mineral Processing and Extractive Metallurgy Review*.

[2] Alipour E., Majidi M. R., Saadatirad A., Golabi S. M., Alizadeh A. M. (2013). Simultaneous determination of dopamine and uric acid in biological samples on the pretreated pencil graphite electrode. *Electrochimica Acta*.

[3] Gong Z. Q., Sujari A. N. A., Ab Ghani S. (2012). Electrochemical fabrication, characterization and application of carboxylic multi-walled carbon nanotube modified composite pencil graphite electrodes. *Electrochimica Acta*.

[4] Liv L., Nakiboğlu N. (2016). Simple and rapid voltammetric determination of boron in water and steel samples using a pencil graphite electrode. *Turkish Journal of Chemistry*.

[5] Kariuki J. K. (2012). An electrochemical and spectroscopic characterization of pencil graphite electrodes. *Journal of the Electrochemical Society*.

[6] Tavares P. H. C. P., Barbeira P. J. S. (2008). Influence of pencil lead hardness on voltammetric response of graphite reinforcement carbon electrodes. *Journal of Applied Electrochemistry*.

[7] http://pencils.com/hb-graphite-grading-scale/

[8] Aoki K., Okamoto T., Kaneko H., Nozaki K., Negishi A. (1989). Applicability of graphite reinforcement carbon used as the lead of a mechanical pencil to voltammetric electrodes. *Journal of Electroanalytical Chemistry*.

[9] Vishnu N., Kumar A. S. (2015). A preanodized 6B-pencil graphite as an efficient electrochemical sensor for mono-phenolic preservatives (phenol and *meta*-cresol) in insulin formulations. *Analytical Methods*.

[10] David I. G., Bizgan A.-M. C., Popa D. E. (2015). Rapid determination of total polyphenolic content in tea samples based on caffeic acid voltammetric behaviour on a disposable graphite electrode. *Food Chemistry*.

[11] David I. G., Buleandră M., Popa D. E. (2016). Voltammetric determination of polyphenolic content as rosmarinic acid equivalent in tea samples using pencil graphite electrodes. *Journal of Food Science and Technology*.

[12] Kariuki J., Ervin E., Olafson C. (2015). Development of a novel, low-cost, disposable wooden pencil graphite electrode for use in the determination of antioxidants and other biological compounds. *Sensors*.

[13] Sharma V. K., Jelen F., Trnkova L. (2015). Functionalized solid electrodes for electrochemical biosensing of purine nucleobases and their analogues: a review. *Sensors (Switzerland)*.

[14] Kawde A., Baig N., Sajid M. (2016). Graphite pencil electrodes as electrochemical sensors for environmental analysis: a review of features, developments, and applications. *RSC Adv.*.

[15] Perdicakis M., Aubriet H., Walcarius A. (2004). Use of a commercially available wood-free resin pencil as convenient electrode for the 'Voltammetry of Microparticles' technique. *Electroanalysis*.

[16] Wang J., Kawde A.-N., Sahlin E. (2000). Renewable pencil electrodes for highly sensitive stripping potentiometric measurements of DNA and RNA. *Analyst*.

[17] Kaneko H., Yamada M., Aoki K. (1990). Determination of dopamine in solution of ascorbic acid at graphite-reinforcement carbon electrodes by differential pulse voltammetry. *Analytical Sciences*.

[18] Vestergaard M., Kerman K., Tamiya E. (2005). An electrochemical approach for detecting copper-chelating properties of flavonoids using disposable pencil graphite electrodes: possible implications in copper-mediated illnesses. *Analytica Chimica Acta*.

[19] Buratti S., Scampicchio M., Giovanelli G., Mannino S. (2008). A low-cost and low-tech electrochemical flow system for the evaluation of total phenolic content and antioxidant power of tea infusions. *Talanta*.

[20] Rizk M., El-Alamin M. M. A., Hendawy H. A. M., Moawad M. I. (2016). Highly sensitive differential pulse and square wave voltammetric methods for determination of strontium ranelate in bulk and pharmaceutical dosage form. *Electroanalysis*.

[21] Özcan A., Şahin Y. (2009). Selective and sensitive voltammetric determination of dopamine in blood by electrochemically treated pencil graphite electrodes. *Electroanalysis*.

[22] Saraji M., Hajialiakbari Bidgoli A. A., Ensafi A. A., Heydari-Bafrooei E., Farajmand B. (2013). Highly sensitive determination of chlorpromazine by electrochemically treated pencil graphite fiber as both solid-phase microextraction fiber and working electrode for use in voltammetry method. *Analytical Methods*.

[23] Bond A. M., Mahon P. J., Schiewe J., Vicente-Beckett V. (1997). An inexpensive and renewable pencil: electrode for use in field-based stripping voltammetry. *Analytica Chimica Acta*.

[24] Aziz M. A., Kawde A.-N. (2013). Nanomolar amperometric sensing of hydrogen peroxide using a graphite pencil electrode modified with palladium nanoparticles. *Microchimica Acta*.

[25] Yardım Y., Şentürk Z. (2013). Electrochemical evaluation and adsorptive stripping voltammetric determination of capsaicin or dihydrocapsaicin on a disposable pencil graphite electrode. *Talanta*.

[26] Gowda J. I., Nandibewoor S. T. (2014). Electrochemical characterization and determination of paclitaxel drug using graphite pencil electrode. *Electrochimica Acta*.

[27] Dilgin D. G., Karakaya S. (2016). Differential pulse voltammetric determination of acyclovir in pharmaceutical preparations using a pencil graphite electrode. *Materials Science and Engineering C*.

[28] Pokpas K., Zbeda S., Jahed N., Mohamed N., Baker P. G., Iwuoha E. I. (2014). Electrochemically reduced graphene oxide pencil-graphite in situ plated bismuth-film electrode for the determination of trace metals by anodic stripping voltammetry. *International Journal of Electrochemical Science*.

[29] Prasad B. B., Madhuri R., Tiwari M. P., Sharma P. S. (2010). Electrochemical sensor for folic acid based on a hyperbranched molecularly imprinted polymer-immobilized sol-gel-modified pencil graphite electrode. *Sensors and Actuators, B: Chemical*.

[30] Santhiago M., Henry C. S., Kubota L. T. (2014). Low cost, simple three dimensional electrochemical paper-based analytical device for determination of p-nitrophenol. *Electrochimica Acta*.

[31] Naik K. M., Ashi C. R., Nandibewoor S. T. (2015). Anodic voltammetric behavior of hydroxyurea and its electroanalytical determination in pharmaceutical dosage form and urine. *Journal of Electroanalytical Chemistry*.

[32] Abdul Aziz M., Kawde A.-N. (2013). Gold nanoparticle-modified graphite pencil electrode for the high-sensitivity detection of hydrazine. *Talanta*.

[33] Akanda M. R., Sohail M., Aziz M. A., Kawde A.-N. (2016). Recent advances in nanomaterial-modified pencil graphite electrodes for electroanalysis. *Electroanalysis*.

[34] Özcan L., Şahin Y., Türk H. (2008). Non-enzymatic glucose biosensor based on overoxidized polypyrrole nanofiber electrode modified with cobalt(II) phthalocyanine tetrasulfonate. *Biosensors and Bioelectronics*.

[35] Gholivand M. B., Khodadadian M., Bahrami G. (2015). Molecularly imprinted polymer preconcentration and flow injection amperometric determination of 4-nitrophenol in water. *Analytical Letters*.

[36] Sağlam Ö., Dilgin D. G., Ertek B., Dilgin Y. (2016). Differential pulse voltammetric determination of eugenol at a pencil graphite electrode. *Materials Science and Engineering C*.

[37] Dede E., Sağlam Ö., Dilgin Y. (2014). Sensitive voltammetric determination of niclosamide at a disposable pencil graphite electrode. *Electrochimica Acta*.

[38] Aoki K., Kobayashi A., Kato N. (1990). Amperometric determination of ozone in water at disposable graphite reinforcement carbon electrodes. *Electroanalysis*.

[39] Kawde A.-N. (2016). Electroanalytical determination of heavy metals in drinking waters in the eastern province of Saudi Arabia. *Desalination and Water Treatment*.

[40] Masawat P., Liawruangrath S., Vaneesorn Y., Liawruangrath B. (2002). Design and fabrication of a low-cost flow-through cell for the determination of acetaminophen in pharmaceutical formulations by flow injection cyclic voltammetry. *Talanta*.

[41] Rubianes M. D., Rivas G. A. (2003). Amperometric quantification of dopamine using different carbon electrodes modified with a melanin-type polymer. *Analytical Letters*.

[42] King D., Friend J., Kariuki J. (2010). Measuring vitamin c content of commercial orange juice using a pencil lead electrode. *Journal of Chemical Education*.

[43] Aladag N., Trnkova L., Kourilova A., Ozsoz M., Jelen F. (2010). Voltammetric study of aminopurines on pencil graphite electrode in the presence of copper ions. *Electroanalysis*.

[44] David I. G., Florea M., Cracea O. G. (2015). Voltammetric determination of B1 and B6 vitamins using a pencil graphite electrode. *Chemical Papers*.

[45] David I. G., Popa D. E., Calin A.-A., Buleandră M., Iorgulescu E.-E. (2016). Voltammetric determination of famotidine on a disposable pencil graphite electrode. *Turkish Journal of Chemistry*.

[46] David I. G., Popa D. E., Buleandra M., Moldovan Z., Iorgulescu E. E., Badea I. A. (2016). Cheap pencil graphite electrodes for rapid voltammetric determination of chlorogenic acid in dietary supplements. *Anal. Methods*.

[47] Rana A., Kawde A.-N. (2016). Novel electrochemically treated graphite pencil electrode surfaces for the determination of trace *α*-naphthol in water samples. *Journal of the Chinese Chemical Society*.

[48] Hendawy H. A., El Kady E. F., El Qudaby H. M., Omran M. A. (2016). Highly sensitive voltammetric determination of dantrolene sodium in pure form, pharmaceuticals, human breast milk and urine at pencil graphite and glassy carbon electrodes. *Indo American Journal of Pharmaceutical Sciences*.

[49] Temerk Y. M., Ibrahim H. S. M., Schuhmann W. (2016). Square wave cathodic adsorptive stripping voltammetric determination of the anticancer drugs flutamide and irinotecan in biological fluids using renewable pencil graphite electrodes. *Electroanalysis*.

[50] David I. G., Badea I. A., Radu G. L. (2013). Disposable carbon electrodes as an alternative for the direct voltammetric determination of alkyl phenols from water samples. *Turkish Journal of Chemistry*.

[51] Vu D. L., Ertek B., Dilgin Y., Červenka L. (2015). Voltammetric determination of tannic acid in beverages using pencil graphite electrode. *Czech Journal of Food Sciences*.

[52] Rizk M., Hendawy H. A. M., Abou El-Alamin M. M., Moawad M. I. (2015). Sensitive anodic voltammetric determination of methylergometrine maleate in bulk and pharmaceutical dosage forms using differential pulse voltammetry. *Journal of Electroanalytical Chemistry*.

[53] Levent A., Yardim Y., Senturk Z. (2009). Voltammetric behavior of nicotine at pencil graphite electrode and its enhancement determination in the presence of anionic surfactant. *Electrochimica Acta*.

[54] Özcan A. (2014). Synergistic effect of lithium perchlorate and sodium hydroxide in the preparation of electrochemically treated pencil graphite electrodes for selective and sensitive bisphenol a detection in water samples. *Electroanalysis*.

[55] Serrano N., Alberich A., Trnkova L. (2012). Oxidation of 6-benzylaminopurine-copper(I) complex on pencil graphite electrode. *Electroanalysis*.

[56] Eksin E., Congur G., Erdem A. (2015). Electrochemical assay for determination of gluten in flour samples. *Food Chemistry*.

[57] Özcan A., Şahin Y. (2011). A novel approach for the determination of paracetamol based on the reduction of N-acetyl-p-benzoquinoneimine formed on the electrochemically treated pencil graphite electrode. *Analytica Chimica Acta*.

[58] Elqudaby H. M., Hendawy H. A. M., Souaya E. R., Mohamed G. G., Eldin G. M. G. (2015). Sensitive electrochemical behavior of cinchocaine hydrochloride at activated glassy carbon and graphite pencil electrodes. *International Journal of Pharmaceutical Analysis*.

[59] Özkorucuklu S. P., Şahin Y., Alsancak G. (2008). Voltammetric behaviour of sulfamethoxazole on electropolymerized- molecularly imprinted overoxidized polypyrrole. *Sensors*.

[60] Rezaei B., Khalili Boroujeni M., Ensafi A. A. (2014). Caffeine electrochemical sensor using imprinted film as recognition element based on polypyrrole, sol-gel, and gold nanoparticles hybrid nanocomposite modified pencil graphite electrode. *Biosensors and Bioelectronics*.

[61] Rezaei B., Lotfi-Forushani H., Ensafi A. A. (2014). Modified Au nanoparticles-imprinted sol-gel, multiwall carbon nanotubes pencil graphite electrode used as a sensor for ranitidine determination. *Materials Science and Engineering C*.

[62] Bindewald E. H., Bergamini M. F., Marcolino-Jr L. H. (2013). Disposable solid-state sensor based on polypyrrole films doped for potentiometric determination of dipyrone in human urine and pharmaceuticals products. *Electroanalysis*.

[63] Ang J. Q., Li S. F. Y. (2012). Novel sensor for simultaneous determination of K^+^ and Na^+^ using Prussian blue pencil graphite electrode. *Sensors and Actuators, B: Chemical*.

[64] Rehacek V., Hotovy I., Vojs M., Mika F. (2008). Bismuth film electrodes for heavy metals determination. *Microsystem Technologies*.

[65] Prasad B. B., Madhuri R., Tiwari M. P., Sharma P. S. (2010). Imprinting molecular recognition sites on multiwalled carbon nanotubes surface for electrochemical detection of insulin in real samples. *Electrochimica Acta*.

[66] Prasad B. B., Pandey I. (2013). Metal incorporated molecularly imprinted polymer-based electrochemical sensor for enantio-selective analysis of pyroglutamic acid isomers. *Sensors and Actuators, B: Chemical*.

[67] Prasad B. B., Kumar D., Madhuri R., Tiwari M. P. (2011). Metal ion mediated imprinting for electrochemical enantioselective sensing of l-histidine at trace level. *Biosensors and Bioelectronics*.

[68] Cantalapiedra A., Gismera M. J., Procopio J. R., Sevilla M. T. (2015). Electrochemical sensor based on polystyrene sulfonate-carbon nanopowders composite for Cu (II) determination. *Talanta*.

[69] Rad A. S. (2011). Vitamin C determination in human plasma using an electro-activated pencil graphite electrode. *Arabian Journal for Science and Engineering*.

[70] Bund A., Dittmann J., Lordkipanidze D., Schwitzgebel G. (1996). A simple and versatile PSA system for heavy metal determinations. *Fresenius' Journal of Analytical Chemistry*.

[71] Rouhollahi A., Kouchaki M., Seidi S. (2016). Electrically stimulated liquid phase microextraction combined with differential pulse voltammetry: a new and efficient design for in situ determination of clozapine from complicated matrices. *RSC Advances*.

[72] Hsieh B. C., Cheng T. J., Shih S. H., Chen R. L. C. (2011). Pencil lead microelectrode and the application on cell dielectrophoresis. *Electrochimica Acta*.

[73] Honeychurch K. C. (2015). The voltammetric behaviour of lead at a hand drawn pencil electrode and its trace determination in water by stripping voltammetry. *Analytical Methods*.

[74] Li W., Qian D., Li Y., Bao N., Gu H., Yu C. (2016). Fully-drawn pencil-on-paper sensors for electroanalysis of dopamine. *Journal of Electroanalytical Chemistry*.

[75] Foster C. W., Brownson D. A. C., Ruas De Souza A. P. (2016). Pencil it in: pencil drawn electrochemical sensing platforms. *Analyst*.

[76] Dossi N., Toniolo R., Impellizzieri F., Bontempelli G. (2014). Doped pencil leads for drawing modified electrodes on paper-based electrochemical devices. *Journal of Electroanalytical Chemistry*.

[77] Dossi N., Toniolo R., Terzi F., Impellizzieri F., Bontempelli G. (2014). Pencil leads doped with electrochemically deposited Ag and AgCl for drawing reference electrodes on paper-based electrochemical devices. *Electrochimica Acta*.

[78] Witkowska Nery E., Guimarães J. A., Kubota L. T. (2015). Paper-based electronic tongue. *Electroanalysis*.

[79] Santhiago M., Kubota L. T. (2013). A new approach for paper-based analytical devices with electrochemical detection based on graphite pencil electrodes. *Sensors and Actuators B: Chemical*.

[80] Oh J.-M., Chow K.-F. (2015). Recent developments in electrochemical paper-based analytical devices. *Analytical Methods*.

[81] Zhang D., Ye K., Yin J., Cheng K., Cao D., Wang G. (2014). Low-cost and binder-free, paper-based cobalt electrode for sodium borohydride electro-oxidation. *New Journal of Chemistry*.

[82] Kurra N., Kulkarni G. U. (2013). Pencil-on-paper: electronic devices. *Lab on a Chip*.

[83] Kurra N., Dutta D., Kulkarni G. U. (2013). Field effect transistors and RC filters from pencil-trace on paper. *Physical Chemistry Chemical Physics*.

[84] Yao B., Yuan L., Xiao X. (2013). Paper-based solid-state supercapacitors with pencil-drawing graphite/polyaniline networks hybrid electrodes. *Nano Energy*.

[85] He J., Luo M., Hu L. (2014). Flexible lead sulfide colloidal quantum dot photodetector using pencil graphite electrodes on paper substrates. *Journal of Alloys and Compounds*.

[86] Blum D., Leyffer W., Holze R. (1996). Pencil-Leads as new electrodes for abrasive stripping voltammetry. *Electroanalysis*.

[87] Kakizaki T., Hasebe K. (1998). Potentiometric stripping determination of heavy metals using a graphite-reinforcement carbon vibrating electrode. *Fresenius' Journal of Analytical Chemistry*.

[88] Doménech-Carbó A., Doménech-Carbó M. T., Peiró-Ronda M. A. (2011). 'One-touch' voltammetry of microparticles for the identification of corrosion products in archaeological lead. *Electroanalysis*.

[89] Doménech-Carbó A., Doménech-Carbó M. T., Peiró-Ronda M. A., Osete-Cortina L. (2011). Electrochemistry and authentication of archaeological lead using voltammetry of microparticles: application to the *Tossal De Sant Miquel* Iberian Plate. *Archaeometry*.

[90] Bagoji A. M., Patil S. M., Nandibewoor S. T. (2016). Electroanalysis of cardioselective beta-adrenoreceptor blocking agent acebutolol by disposable graphite pencil electrodes with detailed redox mechanism. *Cogent Chemistry*.

[91] Supalkova V., Petrek J., Havel L. (2006). Electrochemical sensors for detection of acetylsalicylic acid. *Sensors*.

[92] Ly S. Y., Jung Y. S., Kim M. H., Han I. K., Jung W. W., Kim H. S. (2004). Determination of caffeine using a simple graphite pencil electrode with square-wave anodic stripping voltammetry. *Microchimica Acta*.

[93] Shalaby A., Hassan W. S., Hendawy H. A. M., Ibrahim A. M. (2016). Electrochemical oxidation behavior of itraconazole at different electrodes and its anodic stripping determination in pharmaceuticals and biological fluids. *Journal of Electroanalytical Chemistry*.

[94] Petrek J., Havel L., Petrlova J. (2007). Analysis of salicylic acid in willow barks and branches by an electrochemical method. *Russian Journal of Plant Physiology*.

[95] Krizkova S., Krystofova O., Trnkova L. (2009). Silver(I) ions ultrasensitive detection at carbon electrodes—analysis of waters, tobacco cells and fish tissues. *Sensors*.

[96] Gao W., Song J., Wu N. (2005). Voltammetric behavior and square-wave voltammetric determination of trepibutone at a pencil graphite electrode. *Journal of Electroanalytical Chemistry*.

[97] Ly S. Y., Lee J.-H., Jung D. H. (2010). Radioactive uranium measurement in vivo using a handheld interfaced analyzer. *Environmental Toxicology and Chemistry*.

[98] Gowda J. I., Nandibewoor S. T. (2014). Simultaneous electrochemical determination of 4-aminophenazone and caffeine at electrochemically pre-treated graphite pencil electrode. *Analytical Methods*.

[99] Keskin E., Yardim Y., Şenturk Zühre Z. (2010). Voltammetry of Benzo[a]pyrene in aqueous and nonaqueous media: adsorptive stripping voltammetric determination at pencil graphite electrode. *Electroanalysis*.

[100] Yardim Y., Şentürk Z. (2011). Voltammetric behavior of indole-3-acetic acid and kinetin at pencil-lead graphite electrode and their simultaneous determination in the presence of anionic surfactant. *Turkish Journal of Chemistry*.

[101] Oghli A. H., Alipour E., Asadzadeh M. (2015). Development of a novel voltammetric sensor for the determination of methamphetamine in biological samples on the pretreated pencil graphite electrode. *RSC Advances*.

[102] Alipour E., Gasemlou S. (2012). Easy modification of pencil graphite electrode for discrimination and determination of morphine in biological and street samples. *Analytical Methods*.

[103] Elqudaby H. M., Hendawy H. A., Souaya E. R., Mohamed G. G., Eldin G. M. (2016). Utility of activated glassy carbon and pencil graphite electrodes for voltammetric determination of nalbuphine hydrochloride in pharmaceutical and biological fluids. *International Journal of Electrochemistry*.

[104] Rana A., Kawde A.-N. (2016). Open-circuit electrochemical polymerization for the sensitive detection of phenols. *Electroanalysis*.

[105] Vu D. L., Žabcíková S., Cervenka L., Ertek B., Dilgin Y. (2015). Sensitive voltammetric determination of natural flavonoid quercetin on a disposable graphite lead. *Food Technology and Biotechnology*.

[106] Özcan A., Şahin Y. (2010). Preparation of selective and sensitive electrochemically treated pencil graphite electrodes for the determination of uric acid in urine and blood serum. *Biosensors and Bioelectronics*.

[107] Alipour E., Majidi M. R., Saadatirad A., Golabi S. M. (2012). Determination of uric acid in biological samples on the pretreated pencil graphite electrode. *Analytical Methods*.

[108] Gorcay H., Celik I., Yurdakul E., Sahin Y., Kokten S. (2016). Highly sensitive electrochemical determination of acetaminophen in pharmaceuticals by poly[2, 5-di(2-thiophenyl)-1-p-(tolyl)pyrrole] modified pencil graphite electrode. *IEEE Sensors Journal*.

[109] Saleh G. A., Askal H. F., Refaat I. H., Abdel-Aal F. A. M. (2015). Adsorptive square wave voltammetric determination of acyclovir and its application in a pharmacokinetic study using a novel sensor of *β*-cyclodextrin modified pencil graphite electrode. *Bulletin of the Chemical Society of Japan*.

[110] Nezhadali A., Pirayesh S., Shadmehri R. (2013). Computer-assisted sensor design and analysis of 2-aminobenzimidazole in biological model samples based on electropolymerized-molecularly imprinted polypyrrole modified pencil graphite electrode. *Sensors and Actuators, B: Chemical*.

[111] Prasad B. B., Prasad A., Tiwari M. P. (2013). Highly selective and sensitive analysis of *γ*-aminobutyric acid using a new molecularly imprinted polymer modified at the surface of abrasively immobilized multi-walled carbon nanotubes on pencil graphite electrode. *Electrochimica Acta*.

[112] Ansari R., Mosayebzadeh Z., Mohammad-Khah A. (2014). Fabrication of a solid-state ion selective electrode based on polypyrrole conducting polymer for As (V) ion. *International Journal of Environmental Analytical Chemistry*.

[113] Duy P. K., Sohn J., Chung H. (2016). A mechanical pencil lead-supported carbon nanotube/Au nanodendrite structure as an electrochemical sensor for As(III) detection. *The Analyst*.

[114] Özcan L., Şahin M., Şahin Y. (2008). Electrochemical preparation of a molecularly imprinted polypyrrole-modified pencil graphite electrode for determination of ascorbic acid. *Sensors*.

[115] Pandey I., Jha S. S. (2015). Molecularly imprinted polyaniline-ferrocene-sulfonic acid-Carbon dots modified pencil graphite electrodes for chiral selective sensing of D-Ascorbic acid and L-Ascorbic acid: a clinical biomarker for preeclampsia. *Electrochimica Acta*.

[116] Prasad B. B., Pandey I. (2013). Electrochemically imprinted molecular recognition sites on multiwalled carbon-nanotubes/pencil graphite electrode surface for enantioselective detection of d- and l-aspartic acid. *Electrochimica Acta*.

[117] Prasad B. B., Srivastava A., Tiwari M. P. (2013). Molecularly imprinted polymer-matrix nanocomposite for enantioselective electrochemical sensing of d- and l-aspartic acid. *Materials Science and Engineering C*.

[118] Nezhadali A., Mehri L., Shadmehri R. (2012). Determination of benzimidazole in biological model samples using electropolymerized-molecularly imprinted polypyrrole modified pencil graphite sensor. *Sensors and Actuators, B: Chemical*.

[119] Yaman Y. T., Abaci S. (2016). Sensitive adsorptive voltammetric method for determination of Bisphenol A by gold nanoparticle/polyvinylpyrrolidone-modified pencil graphite electrode. *Sensors (Switzerland)*.

[120] Poorahong S., Thammakhet C., Thavarungkul P., Limbut W., Numnuam A., Kanatharana P. (2012). Amperometric sensor for detection of bisphenol A using a pencil graphite electrode modified with polyaniline nanorods and multiwalled carbon nanotubes. *Microchimica Acta*.

[121] Wee Ling J. L., Khan A., Saad B., Ab Ghani S. (2012). Electro polymerized 4-vinyl pyridine on 2B pencil graphite as ionophore for cadmium (II). *Talanta*.

[122] Ling J. L. W., Ab Ghani S. (2013). Poly(4-vinylpyridine-co-aniline)-modified electrode—synthesis, characterization, and application as cadmium(II) ion sensor. *Journal of Solid State Electrochemistry*.

[123] Arslan E., Çakır S. (2016). Electrochemical fabrication of polyproline modified graphite electrode decorated with Pd-Au bimetallic nanoparticles: application for determination of carminic acid. *Journal of Electroanalytical Chemistry*.

[124] Majidi M. R., Asadpour-Zeynali K., Hafezi B. (2011). Electrocatalytic oxidation and determination of ceftriaxone sodium antibiotic in pharmaceutical samples on a copper hexacyanoferrate nanostructure. *Analytical Methods*.

[125] Yan C., Li J., Meng T. (2016). Selective recognition of ciprofloxacin hydrochloride based on molecular imprinted sensor via electrochemical copolymerization of pyrrole and o-phenylenediamine. *International Journal of Electrochemical Science*.

[126] Koirala K., Sevilla F. B., Santos J. H. (2016). Biomimetic potentiometric sensor for chlorogenic acid based on electrosynthesized polypyrrole. *Sensors and Actuators, B: Chemical*.

[127] Uygun Z. O., Dilgin Y. (2013). A novel impedimetric sensor based on molecularly imprinted polypyrrole modified pencil graphite electrode for trace level determination of chlorpyrifos. *Sensors and Actuators, B: Chemical*.

[128] Prasad B. B., Fatma S. (2016). Electrochemical sensing of ultra trace copper(II) by alga-OMNiIIP modified pencil graphite electrode. *Sensors and Actuators, B: Chemical*.

[129] Ansari R., Delavar A. F., Aliakbar A., Mohammad-Khah A. (2012). Solid-state Cu (II) ion-selective electrode based on polyaniline-conducting polymer film doped with copper carmoisine dye complex. *Journal of Solid State Electrochemistry*.

[130] Ansari R., Mosayebzadeh Z., Arvand M., Mohammad-khah A. (2013). A potentiometric solid state copper electrode based on nanostructure polypyrrole conducting polymer film doped with 5-sulfosalicylic acid. *Journal of Nanostructure in Chemistry*.

[131] Mosayebzadeh Z., Ansari R., Mohammad-khah A., Arvand M. (2013). Electrochemical preparation of a copper ion selective electrode based on polypyrrole conducting polymer doped with Ponceau 4R azo dye. *Analytical and Bioanalytical Electrochemistry*.

[132] Majidi M. R., Asadpour-Zeynali K., Hafezi B. (2010). Sensing L-cysteine in urine using a pencil graphite electrode modified with a copper hexacyanoferrate nanostructure. *Microchimica Acta*.

[133] Rezaei B., Irannejad N., Ensafi A. A., Dinari M. (2016). Application of modified carbon quantum dots/multiwall carbon nanotubes/pencil graphite electrode for electrochemical determination of dextromethorphan. *IEEE Sensors Journal*.

[134] Dehghanzade M., Alipour E. (2016). Voltammetric determination of diazepam using a bismuth modified pencil graphite electrode. *Analytical Methods*.

[135] Fard G. P., Alipour E., Ali Sabzi R. E. (2016). Modification of a disposable pencil graphite electrode with multiwalled carbon nanotubes: application to electrochemical determination of diclofenac sodium in some pharmaceutical and biological samples. *Analytical Methods*.

[136] Nezhadali A., Senobari S., Mojarrab M. (2016). 1,4-dihydroxyanthraquinone electrochemical sensor based on molecularly imprinted polymer using multi-walled carbon nanotubes and multivariate optimization method. *Talanta*.

[137] Chandra U., Kumara Swamy B. E., Gilbert O., Sherigara B. S. (2011). Voltammetric detection of dopamine in presence of ascorbic acid and uric acid at poly (xylenol orange) film-coated graphite pencil electrode. *International Journal of Electrochemistry*.

[138] Chandra U., Kumara Swamy B. E., Gilbert O. (2010). Poly(amaranth) film based sensor for resolution of dopamine in the presence of uric acid: a voltammetric study. *Chinese Chemical Letters*.

[139] Chandra U., Swamy B. E. K., Gilbert O., Sherigara B. S. (2011). Poly (Naphthol Green B) film based sensor for resolution of dopamine in the presence of uric acid: a voltammetric study. *Analytical Methods*.

[140] Kalachar H. C. B., Basavanna S., Viswanatha R., Naik Y. A., Raj D. A., Sudha P. N. (2011). Electrochemical determination of l-dopa in *Mucuna pruriens* seeds, leaves and commercial siddha product using gold modified pencil graphite electrode. *Electroanalysis*.

[141] Prasad B. B., Prasad A., Tiwari M. P., Madhuri R. (2013). Multiwalled carbon nanotubes bearing 'terminal monomeric unit' for the fabrication of epinephrine imprinted polymer-based electrochemical sensor. *Biosensors and Bioelectronics*.

[142] Hao G., Zheng D., Gan T., Hu C., Hu S. (2011). Development and application of estradiol sensor based on layer-by-layer assembling technique. *Journal of Experimental Nanoscience*.

[143] Patra S., Roy E., Madhuri R., Sharma P. K. (2015). An imprinted Ag@CdS core shell nanoparticle based optical-electrochemical dual probe for trace level recognition of ferritin. *Biosensors and Bioelectronics*.

[144] Izadyar A., Arachchige D. R., Cornwell H., Hershberger J. C. (2016). Ion transfer stripping voltammetry for the detection of nanomolar levels of fluoxetine, citalopram, and sertraline in tap and river water samples. *Sensors and Actuators, B: Chemical*.

[145] Pourbeyram S., Mehdizadeh K. (2016). Nonenzymatic glucose sensor based on disposable pencil graphite electrode modified by copper nanoparticles. *Journal of Food and Drug Analysis*.

[146] Prasad B. B., Jauhari D., Tiwari M. P. (2014). Doubly imprinted polymer nanofilm-modified electrochemical sensor for ultra-trace simultaneous analysis of glyphosate and glufosinate. *Biosensors and Bioelectronics*.

[147] Pokpas K., Jahed N., Tovide O., Baker P. G., Iwuoha E. I. (2014). Nafion-graphene nanocomposite in situ plated bismuth-film electrodes on pencil graphite substrates for the determination of trace heavy metals by anodic stripping voltammetry. *International Journal of Electrochemical Science*.

[148] Prasad B. B., Prasad A., Tiwari M. P. (2013). Quantum dots-multiwalled carbon nanotubes nanoconjugate-modified pencil graphite electrode for ultratrace analysis of hemoglobin in dilute human blood samples. *Talanta*.

[149] Heydari H., Gholivand M. B., Abdolmaleki A. (2016). Cyclic voltammetry deposition of copper nanostructure on MWCNTs modified pencil graphite electrode: an ultra-sensitive hydrazine sensor. *Materials Science and Engineering C*.

[150] Nezhadali A., Mojarrab M. (2014). Computational study and multivariate optimization of hydrochlorothiazide analysis using molecularly imprinted polymer electrochemical sensor based on carbon nanotube/polypyrrole film. *Sensors and Actuators, B: Chemical*.

[151] Zhang J., Zheng J. (2015). An enzyme-free hydrogen peroxide sensor based on Ag/FeOOH nanocomposites. *Analytical Methods*.

[152] Kawde A.-N., Aziz M., Baig N., Temerk Y. (2015). A facile fabrication of platinum nanoparticle-modified graphite pencil electrode for highly sensitive detection of hydrogen peroxide. *Journal of Electroanalytical Chemistry*.

[153] Gholami M., Ghasemi A.-M., Loghavi M. M., Behkami S., Ahamdi-Dokht-Faraghe A. (2013). Preparation of a miniaturised iodide ion selective sensor using polypyrrole and pencil lead: effect of double-coating, electropolymerisation time, and current density. *Chemical Papers*.

[154] Rezaei B., Boroujeni M. K., Ensafi A. A. (2014). A novel electrochemical nanocomposite imprinted sensor for the determination of lorazepam based on modified polypyrrole@sol-gel@gold nanoparticles/pencil graphite electrode. *Electrochimica Acta*.

[155] Temerk Y., Ibrahim M., Ibrahim H., Kotb M. (2016). Adsorptive stripping voltammetric determination of anticancer drug lomustine in biological fluids using in situ mercury film coated graphite pencil electrode. *Journal of Electroanalytical Chemistry*.

[156] Mosayebzadeh Z., Ansari R., Arvand M. (2014). Preparation of a solid-state ion-selective electrode based on polypyrrole conducting polymer for magnesium ion. *Journal of the Iranian Chemical Society*.

[157] Kumar S., Karfa P., Patra S., Madhuri R., Sharma P. K. (2016). Molecularly imprinted star polymer-modified superparamagnetic iron oxide nanoparticle for trace level sensing and separation of mancozeb. *RSC Advances*.

[158] Alipour E., Majidi M. R., Hoseindokht O. (2015). Development of simple electrochemical sensor for selective determination of methadone in biological samples using multi-walled carbon nanotubes modified pencil graphite electrode. *Journal of the Chinese Chemical Society*.

[159] Prasad B. B., Pandey I., Srivastava A., Kumar D., Tiwari M. P. (2013). Multiwalled carbon nanotubes-based pencil graphite electrode modified with an electrosynthesized molecularly imprinted nanofilm for electrochemical sensing of methionine enantiomers. *Sensors and Actuators B: Chemical*.

[160] Nezhadali A., Mojarrab M. (2016). Computational design and multivariate optimization of an electrochemical metoprolol sensor based on molecular imprinting in combination with carbon nanotubes. *Analytica Chimica Acta*.

[161] Rezaei B., Foroughi-Dehnavi S., Ensafi A. A. (2015). Fabrication of electrochemical sensor based on molecularly imprinted polymer and nanoparticles for determination trace amounts of morphine. *Ionics*.

[162] Bendikov T. A., Harmon T. C. (2005). A sensitive nitrate ion-selective electrode from a pencil lead. An analytical laboratory experiment. *Journal of Chemical Education*.

[163] Dagcı K., Alanyalıoğlu M. (2013). Electrochemical preparation of polymeric films of pyronin Y and its electrolcatalytic properties for amperometric detection of nitrite. *Journal of Electroanalytical Chemistry*.

[164] Kuralay F., Dumangöz M., Tunç S. (2015). Polymer/carbon nanotubes coated graphite surfaces for highly sensitive nitrite detection. *Talanta*.

[165] Kawde A.-N., Aziz M. A. (2014). Porous copper-modified graphite pencil electrode for the amperometric detection of 4-nitrophenol. *Electroanalysis*.

[166] Asadpour-Zeynali K., Najafi-Marandi P. (2011). Bismuth modified disposable pencil-lead electrode for simultaneous determination of 2-nitrophenol and 4-nitrophenol by net analyte signal standard addition method. *Electroanalysis*.

[167] Noronha B. V., Bindewald E. H., De Oliveira M. C., Papi M. A. P., Bergamini M. F., Marcolino-Jr L. H. (2014). Potentiometric determination of pantoprazole using an ion-selective sensor based on polypyrrole doped films. *Materials Science and Engineering C*.

[168] Özcan L., Şahin Y. (2007). Determination of paracetamol based on electropolymerized-molecularly imprinted polypyrrole modified pencil graphite electrode. *Sensors and Actuators, B: Chemical*.

[169] Nezhadali A., Rouki Z., Nezhadali M. (2015). Electrochemical preparation of a molecularly imprinted polypyrrole modified pencil graphite electrode for the determination of phenothiazine in model and real biological samples. *Talanta*.

[170] Muti M., Gençdaĝ K., Nacak F. M., Aslan A. (2013). Electrochemical polymerized 5-amino-2-mercapto-1,3,4-thiadiazole modified single use sensors for detection of quercetin. *Colloids and Surfaces B: Biointerfaces*.

[171] Özcan A., Ilkbaş S. (2015). Poly(pyrrole-3-carboxylic acid)-modified pencil graphite electrode for the determination of serotonin in biological samples by adsorptive stripping voltammetry. *Sensors and Actuators, B: Chemical*.

[172] Ochiai L. M., Bindewald E. H., Mengarda P., Marcolino-Junior L. H., Bergamini M. F. (2016). Disposable potentiometric citrate sensor based on polypyrrole-doped films for indirect determination of sildenafil in pharmaceuticals formulations. *Journal of Applied Polymer Science*.

[173] Tadi K. K., Motghare R. V., Ganesh V. (2014). Electrochemical detection of sulfanilamide using pencil graphite electrode based on molecular imprinting technology. *Electroanalysis*.

[174] Nezhadali A., Shadmehri R. (2013). Computer-aided sensor design and analysis of thiocarbohydrazide in biological matrices using electropolymerized-molecularly imprinted polypyrrole modified pencil graphite electrode. *Sensors and Actuators, B: Chemical*.

[175] Prasad B. B., Singh R., Kumar A. (2016). Development of imprinted polyneutral red/electrochemically reduced graphene oxide composite for ultra-trace sensing of 6-thioguanine. *Carbon*.

[176] Nezhadali A., Mojarrab M. (2015). Fabrication of an electrochemical molecularly imprinted polymer triamterene sensor based on multivariate optimization using multi-walled carbon nanotubes. *Journal of Electroanalytical Chemistry*.

[177] Baig N., Kawde A.-N. (2015). A novel, fast and cost effective graphene-modified graphite pencil electrode for trace quantification of l-tyrosine. *Analytical Methods*.

[178] Ansari R., Mosayebzadeh Z. (2014). Construction of a new solid-state U(VI) ion-selective electrode based on polypyrrole conducting polymer. *Journal of Radioanalytical and Nuclear Chemistry*.

[179] Özcan A., Ilkbaş S. (2015). Preparation of poly(3,4-ethylenedioxythiophene) nanofibers modified pencil graphite electrode and investigation of over-oxidation conditions for the selective and sensitive determination of uric acid in body fluids. *Analytica Chimica Acta*.

[180] Saleh G. A., Askal H. F., Refaat I. H., Naggar A. H., Abdel-aal F. A. M. (2016). Adsorptive square wave voltammetric determination of the antiviral drug valacyclovir on a novel sensor of copper microparticles-modified pencil graphite electrode. *Arabian Journal of Chemistry*.

[181] Pala B. B., Vural T., Kuralay F. (2014). Disposable pencil graphite electrode modified with peptide nanotubes for Vitamin B12 analysis. *Applied Surface Science*.

[182] Kuralay F., Vural T., Bayram C., Denkbas E. B., Abaci S. (2011). Carbon nanotube–chitosan modified disposable pencil graphite electrode for Vitamin B_12_ analysis. *Colloids and Surfaces B: Biointerfaces*.

[183] Ansari R., Delavar A. F., Mohammad-Khah A. (2012). Solid-state ion selective electrode based on polypyrrole conducting polymer nanofilm as a new potentiometric sensor for Zn^2+^ ion. *Journal of Solid State Electrochemistry*.

[184] Prasad B. B., Srivastava A., Tiwari M. P. (2013). Highly sensitive and selective hyphenated technique (molecularly imprinted polymer solid-phase microextraction-molecularly imprinted polymer sensor) for ultra trace analysis of aspartic acid enantiomers. *Journal of Chromatography A*.

[185] Ensafi A. A., Khoddami E., Rezaei B. (2013). A combined liquid three-phase micro-extraction and differential pulse voltammetric method for preconcentration and detection of ultra-trace amounts of buprenorphine using a modified pencil electrode. *Talanta*.

[186] Prasad B. B., Srivastava A., Prasad A., Tiwari M. P. (2014). Molecularly imprinted micro solid-phase extraction technique coupled with complementary molecularly imprinted polymer-sensor for ultra trace analysis of epinephrine in real samples. *Colloids and Surfaces B: Biointerfaces*.

[187] Ensafi A. A., Khoddami E., Rezaei B. (2016). Development of a cleanup and electrochemical determination of flutamide using silica thin film pencil graphite electrode functionalized with thiol groups. *Journal of the Iranian Chemical Society*.

[188] Ensafi A. A., Khoddami E., Rezaei B., Jafari-Asl M. (2015). A supported liquid membrane for microextraction of insulin, and its determination with a pencil graphite electrode modified with RuO_2_-graphene oxide. *Microchimica Acta*.

[189] Prasad B. B., Srivastava A., Pandey I., Tiwari M. P. (2013). Electrochemically grown imprinted polybenzidine nanofilm on multiwalled carbon nanotubes anchored pencil graphite fibers for enantioselective micro-solid phase extraction coupled with ultratrace sensing of D- and l-methionine. *Journal of Chromatography B*.

[190] Fakhari A. R., Sahragard A., Ahmar H., Tabani H. (2015). A novel platform sensing based on combination of electromembrane-assisted solid phase microextraction with linear sweep voltammetry for the determination of tramadol. *Journal of Electroanalytical Chemistry*.

[191] Asofiei I., Calinescu I., Trifan A., David I. G., Gavrila A. I. (2016). Microwave-assisted batch extraction of polyphenols from sea buckthorn leaves. *Chemical Engineering Communications*.

[192] Dilgin Y., Kizilkaya B., Ertek B., Eren N., Dilgin D. G. (2012). Amperometric determination of sulfide based on its electrocatalytic oxidation at a pencil graphite electrode modified with quercetin. *Talanta*.

[193] Dilgin Y., Kizilkaya B., Dilgin D. G., Gökçel H. I., Gorton L. (2013). Electrocatalytic oxidation of NADH using a pencil graphite electrode modified with quercetin. *Colloids and Surfaces B: Biointerfaces*.

[194] Navratil R., Pilarova I., Jelen F., Trnkova L. (2013). Comparative voltammetric analysis of adenine and xanthine on a pencil graphite electrode in the presence of copper ions. *International Journal of Electrochemical Science*.

[195] Navratil R., Jelen F., UgurKayran Y., Trnkova L. (2014). A pencil graphite electrode in situ modified by monovalent copper: a promising tool for the determination of methylxanthines. *Electroanalysis*.

[196] Suw Y. L., Min J. K. (2009). Diagnostic assay of chromium (VI) in the ex vivo fluid of the urine of a smoker using a fluorine-doped handmade sensor. *Journal of Clinical Laboratory Analysis*.

[197] Dilgin Y., Kızılkaya B., Ertek B., Işik F., Dilgin D. G. (2012). Electrocatalytic oxidation of sulphide using a pencil graphite electrode modified with hematoxylin. *Sensors and Actuators B: Chemical*.

[198] Kuralay F., Yilmaz E., Uzun L., Denizli A. (2013). Cibacron Blue F3GA modified disposable pencil graphite electrode for the investigation of affinity binding to bovine serum albumin. *Colloids and Surfaces B: Biointerfaces*.

[199] David I. G., Matache M. L., Radu G. L., Ciucu A. A., Pirrone N. (2013). Cheap in situ voltammetric copper determination from freshwater samples. *E3S Web of Conferences*.

[200] Majidi M. R., Asadpour-Zeynali K., Hafezi B. (2011). Fabrication of nanostructured copper thin films at disposable pencil graphite electrode and its application to elecrocatalytic reduction of nitrate. *International Journal of Electrochemical Science*.

[201] Alam M. M., Hasnat M. A., Rashed M. A. (2015). Nitrate detection activity of Cu particles deposited on pencil graphite by fast scan cyclic voltammetry. *Journal of Analytical Chemistry*.

[202] Safardoust-Hojaghan H., Salavati-Niasari M., Motaghedifard M. H., Hosseinpour-Mashkani S. M. (2015). Synthesis of micro sphere-like bismuth nanoparticles by microwave assisted polyol method; designing a novel electrochemical nanosensor for ultra-trace measurement of Pb^2+^ ions. *New Journal of Chemistry*.

[203] Etesami M., Karoonian F. S., Mohamed N. (2013). Electrooxidation of hydroquinone on simply prepared Au-Pt bimetallic nanoparticles. *Science China Chemistry*.

[204] Goudarzi M., Salavati-Niasari M., Bazarganipour M., Motaghedifard M. (2016). Sonochemical synthesis of Tl2O3 nanostructures: supported on multi-walled carbon nanotube modified electrode for monitoring of copper ions. *Journal of Materials Science: Materials in Electronics*.

[205] Vural T., Kuralay F., Bayram C., Abaci S., Denkbas E. B. (2010). Preparation and physical/electrochemical characterization of carbon nanotube-chitosan modified pencil graphite electrode. *Applied Surface Science*.

[206] Ouyang J., Li Z.-Q., Zhang J. (2014). A rapid and sensitive method for hydroxyl radical detection on a microfluidic chip using an N-doped porous carbon nanofiber modified pencil graphite electrode. *Analyst*.

[207] Gao W., Song J. (2009). Polyaniline film based amperometric pH sensor using a novel electrochemical measurement system. *Electroanalysis*.

[208] Gao W., Wu N., Song J. (2012). A pH-controllable electrochemical molecule switch employing a new electrochemical measurement system as switching transducer. *Journal of Solid State Electrochemistry*.

[209] Jin J., Hiroi T., Sato K., Miwa T., Takeuchi T. (2002). Use of disposable GRC electrodes for the detection of phenol and chlorophenols in liquid chromatography. *Analytical Sciences*.

[210] Karaca E., Pekmez N. Ö., Pekmez K. (2014). Galvanostatic deposition of polypyrrole in the presence of tartaric acid for electrochemical supercapacitor. *Electrochimica Acta*.

[211] Arslan A., Hür E. (2012). Supercapacitor applications of polyaniline and poly(n-methylaniline) coated pencil graphite electrode. *International Journal of Electrochemical Science*.

[212] Arslan A., Hur E. (2014). Electrochemical storage properties of polyaniline-, poly(N-methylaniline)-, and poly(N-ethylaniline)-coated pencil graphite electrodes. *Chemical Papers*.

[213] Hur E., Arslan A. (2014). Cobalt ion-doped polyaniline, poly(N-methylaniline), and poly(N-ethylaniline): electrosynthesis and characterisation using electrochemical methods in acidic solutions. *Chemical Papers*.

[214] Hür E., Varol G. A., Arslan A. (2013). The study of polythiophene, poly(3-methylthiophene) and poly(3,4-ethylenedioxythiophene) on pencil graphite electrode as an electrode active material for supercapacitor applications. *Synthetic Metals*.

[215] Hür E., Arslan A. (2014). New electrode active materials for supercapacitors: pencil graphite electrode coated with cobalt ion doped poly(3-methylthiophene) and poly(3,4-ethylenedioxythiophene). *Synthetic Metals*.

[216] Çelik B., Çelik I., Dolaş H. (2014). Electrochemical synthesis, characterization and capacitive properties of novel thiophene based conjugated polymer. *Reactive and Functional Polymers*.

[217] Nasir A., Kausar A., Younus A. (2015). Polymer/Graphite nanocomposites: physical features, fabrication and current relevance. *Polymer—Plastics Technology and Engineering*.

[218] Majidi M. R., Asadpour-Zeynali K., Hafezi B. (2009). Reaction and nucleation mechanisms of copper electrodeposition on disposable pencil graphite electrode. *Electrochimica Acta*.

[219] Rezaei M., Tabaian S. H., Haghshenas D. F. (2012). Nucleation and growth of Pd nanoparticles during electrocrystallization on pencil graphite. *Electrochimica Acta*.

